# *Spongia* Sponges: Unabated Sources of Novel Secondary Metabolites

**DOI:** 10.3390/md22050213

**Published:** 2024-05-07

**Authors:** Qi-Bin Yang, Lin-Fu Liang

**Affiliations:** College of Chemistry and Chemical Engineering, Central South University of Forestry and Technology, Changsha 410004, China

**Keywords:** marine sponges, *Spongia*, secondary metabolites, bioactivities

## Abstract

Marine sponges of the genus *Spongia* have proven to be unabated sources of novel secondary metabolites with remarkable scaffold diversities and significant bioactivities. The discovery of chemical substances from *Spongia* sponges has continued to increase over the last few years. The current work provides an up-to-date literature survey and comprehensive insight into the reported metabolites from the members of the genus *Spongia*, as well as their structural features, biological activities, and structure–activity relationships when available. In this review, 222 metabolites are discussed based on published data from the period from mid-2015 to the beginning of 2024. The compounds are categorized into sesquiterpenes, diterpenes, sesterterpenes, meroterpenes, linear furanoterpenes, steroids, alkaloids, and other miscellaneous substances. The biological effects of these chemical compositions on a vast array of pharmacological assays including cytotoxic, anti-inflammatory, antibacterial, neuroprotective, protein tyrosine phosphatase 1B (PTP1B)-inhibitory, and phytoregulating activities are also presented.

## 1. Introduction

Marine natural products are an enormous source of potentially novel and biologically active compounds [1]. They are frequently developed as lead compounds and drugs [2,3], such as for anticancer [4,5], antimalarial [6], and human immunity purposes [7]. They can also be used as cosmetic ingredients [8], insecticides [9], and environmentally friendly antifoulants [10]. Among the various marine organisms, marine sponges of the order Dictyoceratida have been proven to be a remarkable biochemical warehouse for marine natural products [11,12].

The genus *Spongia* belongs to the family Spongidae of the order Dictyoceratida and comprises three subgenera, *Australospongia*, *Heterofibria*, and *Spongia*, containing 1, 7, and 81 species, respectively. To provide a full-aspect and in-depth view on novel secondary metabolites of *Spongia* sponges, Patrícia Máximo et al. published the first review of this genus entitled ‘‘The role of *Spongia* sp. in the discovery of marine lead compounds’’ in 2016. In the previously mentioned review, the sources, chemical structures, and bioactivities of 306 compounds were described from the sponges of the genus *Spongia* from 1971 to early 2015 [13]. Following the rapid development of marine natural products research, more and more secondary metabolites from *Spongia* sponges have been reported in recent years. As outlined in an extensive literature survey, 222 metabolites were obtained from different species of the genus *Spongia* from mid-2015 to the beginning of 2024. So many compounds reported in such a short period revealed the demand for an up-to-date review.

In this review, the secondary metabolites of *Spongia* sponges displayed a remarkable array of complex molecular architectures, which could be divided into eight groups, including sesquiterpenes, diterpenes, sesterterpenes, meroterpenes, linear furanoterpenes, steroids, alkaloids, and other miscellaneous. And these isolates were subjected to a wide spectrum of bioassays, such as cytotoxic, anti-inflammatory, antibacterial, neuroprotective, PTP1B inhibitory, and phytoregulating activities. Herein, the metabolites of the members of this genus *Spongia*, as well as their structural features, biological activities, and structure–activity relationships, when available, are presented.

## 2. Sesquiterpenes

**Figure 1:**
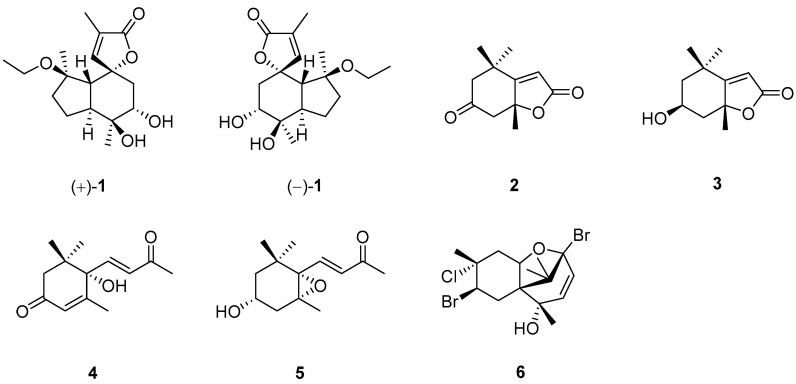
Sesquiterpenes obtained from the genus *Spongia*.

This class of compounds is referred to as sesquiterpenes or norsesquiterpenes without an additional non-terpenoidal subunit (Figure 1).

A study on the Chinese sponge *Spongia* sp. led to the discovery of a pair of new valerenane sesquiterpene enantiomers, (+)- and (−)-spongiterpene [(+)-**1** and (−)-**1**], along with two C_11_ nor-sesquiterpenes, dehydrololiolide (**2**) and loliolide (**3**), and two C_13_ nor-sesquiterpenes, dehydrovomifoliol (**4**) and 3β-hydroxy-5α,6α-epoxy-7-megastimen-9-one (**5**). It might be worth pointing out that quantum chemical ECD calculations were applied to determine the absolute configurations of (+)-**1** and (−)-**1**. The cytotoxic activities of (+)-**1** and (−)-**1** were evaluated against several cancer cells including K562, A549, Hela, HT29, SK-BR-3, Huh7, RBL-2H3 and PC3. However, (+)-**1** and (−)-**1** showed no anti-proliferative effects on these carcinoma cells at 100 μM [14]. An investigation involving the Red Sea sponge *Spongia* sp. afforded pacifenol (**6**), which is a trihalogenated sesquiterpene with a chamigrane skeleton. This was the first report of pacifenol (**6**) in the sponge. This compound was subjected to several bioassays including cytotoxicity against the Huh7 cell line, antibacterial activity against *Staphylococcus aureus*, and anti-inflammatory activity. However, it was inactive in these biotests [15].

## 3. Diterpenes

**Figure 2:**
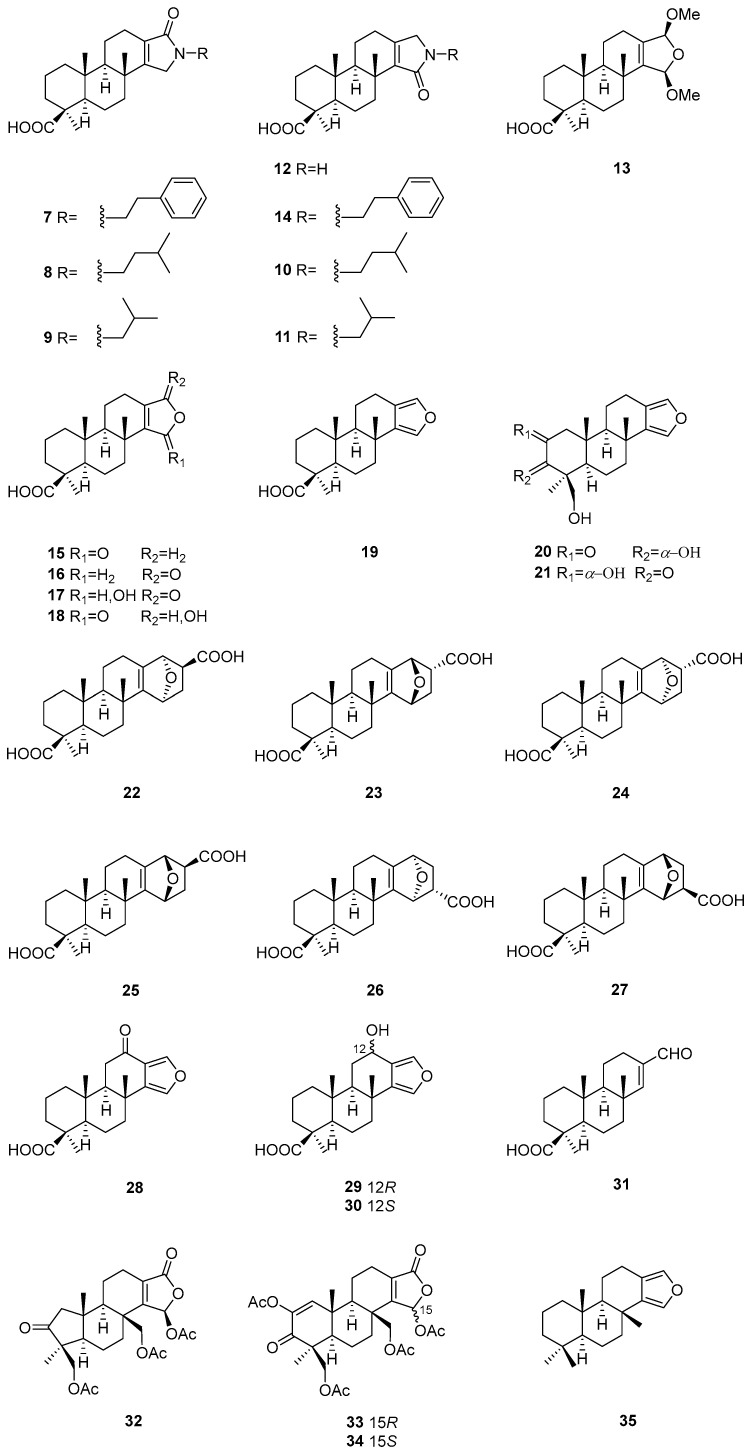

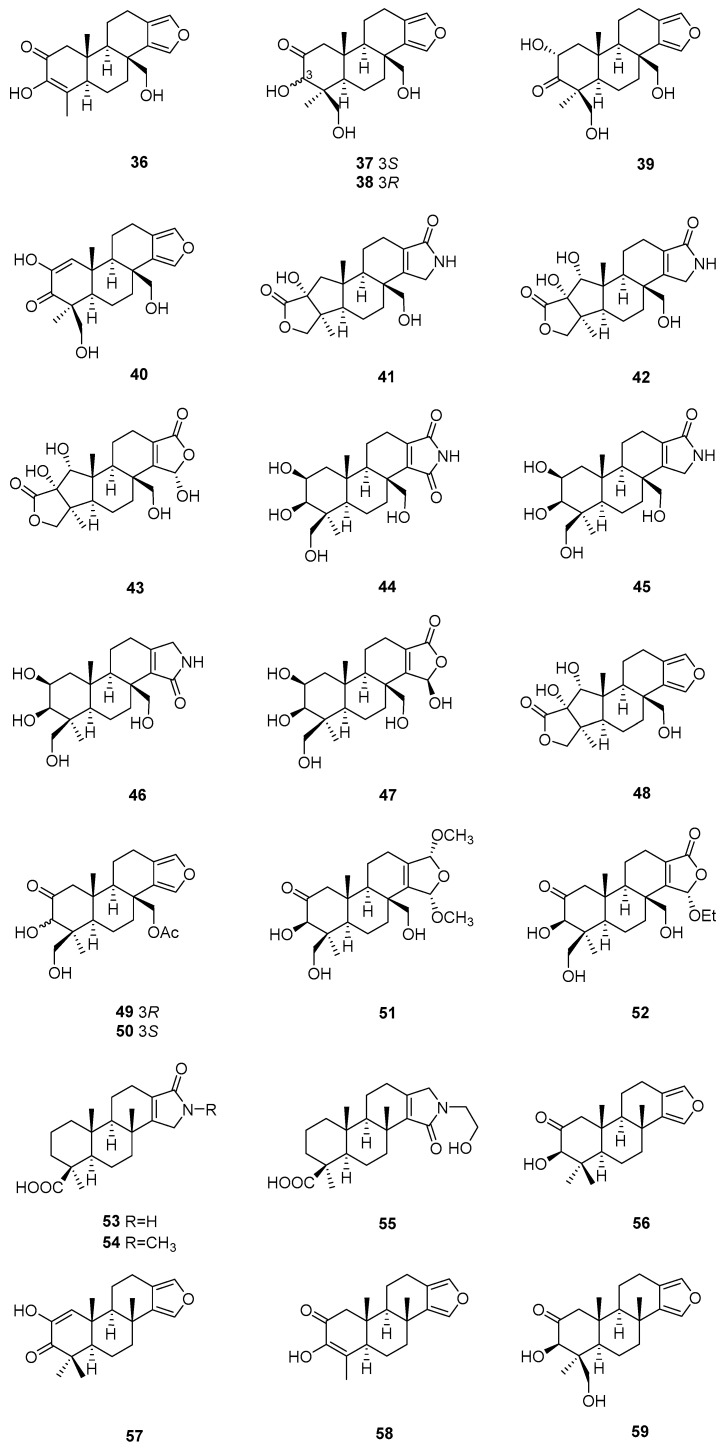

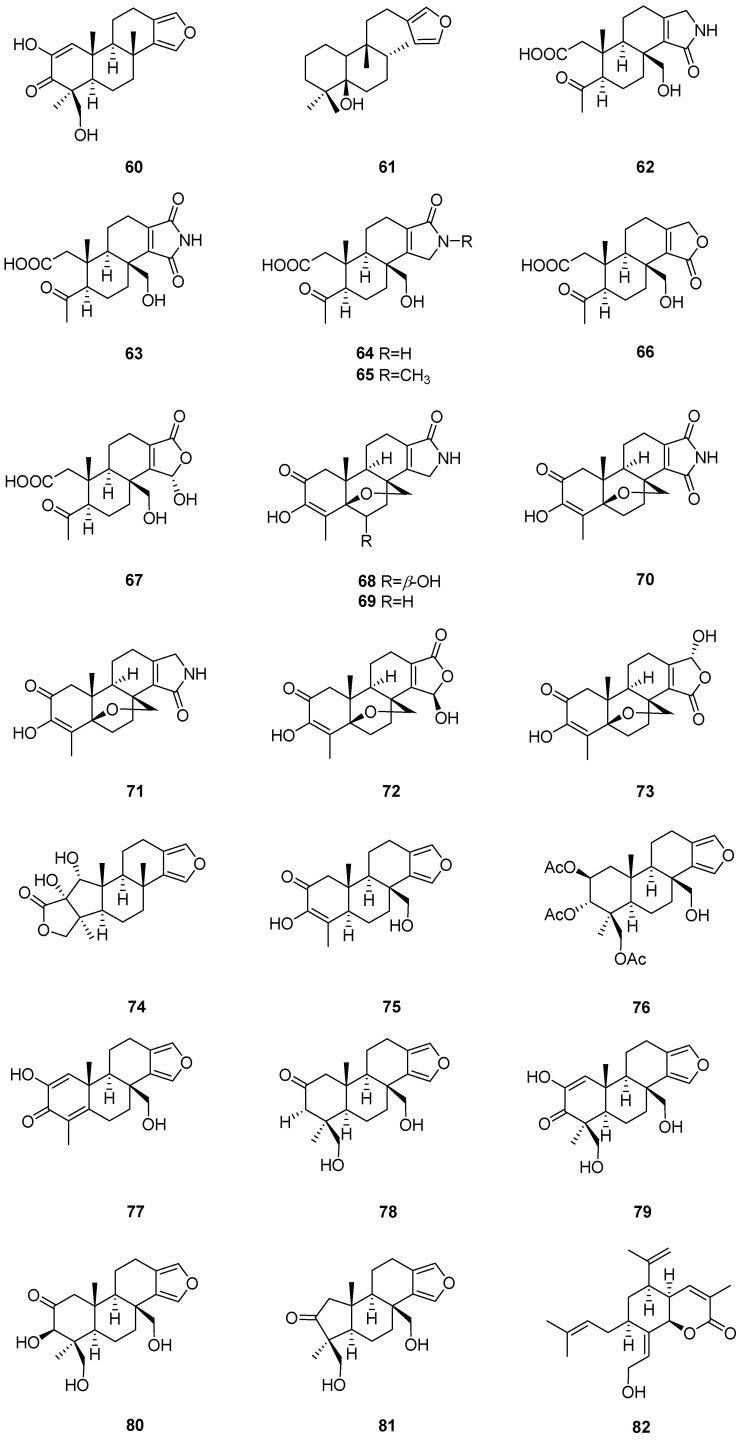

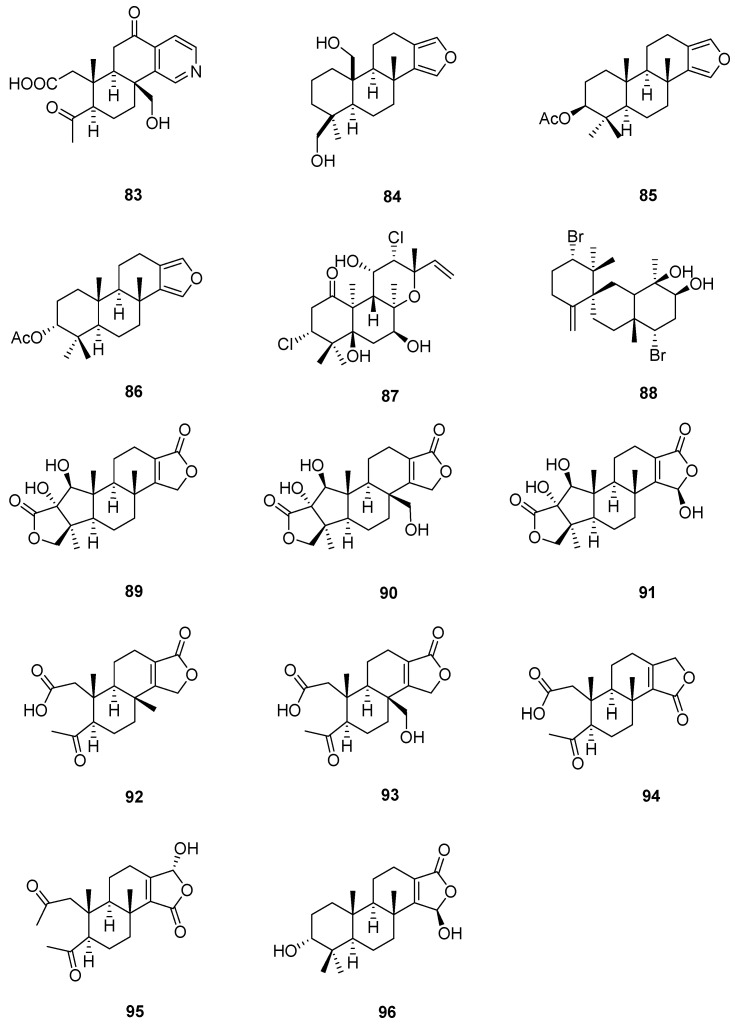
Diterpenes obtained from the genus *Spongia*.

Diterpenes are the most abundant terpenes found in *Spongia* sponges. Of which, spongian is the characteristic type of diterpene and this name reflects the origin of the early examples of this class of diterpene in the sponge genus *Spongia* [16]. In this work, most of the spongian diterpenes were tetracyclic, containing a furan ring. In some cases, the furan was replaced with an unsaturated γ-lactone or γ-lactam or a γ-hydroxybutenolide. Especially intriguing members of this group are degraded, ring contracted, or *seco*-diterpenes (Figure 2).

An investigation involving the Indonesian marine sponge *Spongia ceylonensis* disclosed the presence of six new nitrogenous spongian diterpenes, ceylonamides A–F (**7**–**12**), and one new spongian diacetal, 15α,16-dimethoxyspongi-13-en-19-oic acid (**13**), together with eight known terpenes **14**–**21**. These secondary metabolites were evaluated for their inhibitory effects on the RANKL-induced formation of multinuclear osteoclasts in RAW264 macrophages. The results revealed that ceylonamides A (**7**) and B (**8**) exhibited significant inhibitory activities (IC_50_ = 13 and 18 μM, respectively), whereas the IC_50_ values of the other compounds, **9**–**21**, were more than 50 μM. Inspection of the structure–activity relationships of these isolates inferred that the position of the carbonyl group of the γ-lactam ring and bulkiness of the substituent at its nitrogen atom played important roles in the inhibitory properties [17].

Research on another specimen of Indonesian marine sponge *S. ceylonensis* led to the isolation of six new spongian diterpene derivatives, ceylonins A–F (**22**–**27**), as well as the previously reported compound **19**. Interestingly, these new compounds harbored three additional carbons in ring D to generate an ether-bridged bicyclic ring system. The absolute configurations of these newly discovered terpenes were elucidated based on the comparison of the calculated ECD spectra with the corresponding experimental ones. The bicyclic ring system might be derived from the major metabolite **19** and a C_3_ unit (an acrylic acid equivalent) through an intermolecular Diels–Alder reaction, which was experimentally supported by the transformation of **19** and acrylic acid to **22**–**27**. The inhibitory effects of all of the isolates on the RANKL-induced formation of multinuclear osteoclasts in RAW264 macrophages were examined. Among them, **22** significantly inhibited the formation of multinuclear osteoclasts by 70% in a dose-dependent manner without cytotoxicity, followed by **26** (47%), **27** (31%), and **25** (28%). However, the major compound **19** did not inhibit the formation of multinuclear osteoclasts at 50 μM [18]. From the view of structure–activity relationships, the ether-bridged bicyclic ring system was likely a key functionality for the bioactivity. Further purification of the above-mentioned sample afforded three new spongian diterpenes, ceylonins G–I (**28**–**30**), together with known compound *ent*-13-norisocopalen-15-al-18-oic acid (**31**), in addition to the five previously reported ones **15**–**18**. The absolute configurations of these new substances were determined via ECD calculations. In this work, the ubiquitin-specific protease 7 (USP7) inhibitory activities of compounds **7**–**12**, **15**–**19**, and **22**–**31** were evaluated. As a result, only **31** inhibited USP7 with an IC_50_ value of 8.2 μM, while the IC_50_ values of the other compounds were more than 50 μM. The substructure including an aldehyde group at the C-ring in **31** may be important for this inhibition [19].

The South China Sea sponge *Spongia officinalis* afforded eight metabolic components including three new diterpenes 3-nor-spongiolide A (**32**) and spongiolides A (**33**) and B (**34**), along with six related known ones, **35**–**40**. Notably, compound **32** possessed the extremely rare 3-nor-spongian carbon architecture. ECD calculations were employed to assign the absolute configurations of three new substances, **32**–**34**. In the cytotoxic bioassay, none of these isolates exhibited potent cytotoxic activity against HL-60 cell lines [20]. Spongiains A–G (**41**–**47**) were yielded by the Chinese marine sponge *Spongia* sp. Intriguingly, compounds **41**–**43** were the first examples of spongian diterpenes bearing a pentacyclic skeleton composed of a fused 5/5/6/6/5 ring system derived from ring A rearrangement. X-ray diffraction experiments and quantum chemical approaches were used to establish the absolute configurations of new metabolites **41**–**47**. These natural products were inactive against an array of cancer cells (K562, A549, Hela, HT29, SK-BR-3, Huh7, RBL-2H3, and PC3) in the anti-proliferative bioassay. Nevertheless, **42** and **43** were able to promote the proliferation of RBL-2H3 cells in dose-dependent concentrations between 10 and 80 μM [21].

An unprecedented 5,5,6,6,5-pentacyclic diterpene, sponalactone (**48**), two new spongian diterpenes, 17-*O*-acetylepispongiatriol (**49**) and 17-*O*-acetylspongiatriol (**50**), and two new spongian diterpene artifacts, 15α,16α-dimethoxy-15,16-dihydroepispongiatriol (**51**) and 15α-ethoxyepispongiatriol-16(15*H*)-one (**52**), together with previously found metabolites **37**–**39**, were obtained from another specimen of South China Sea sponge *S. officinalis*. The absolute configurations of these new compounds were assigned on the basis of ECD data. In the primary screening, compounds **38** and **48**–**52** displayed moderate inhibition against lipopolysaccharide (LPS)-induced nitric oxide (NO) production in RAW264.7 macrophages with IC_50_ values of 12–32 μM, whereas no inhibitory effect was observed for **39** (IC_50_ > 60 μM). The weaker activities of **39** and **48** in comparison with **38** and **49**−**52** suggested that 2-oxo-3-hydroxycyclohexane was an essential substructure in ring A for inhibitory activity [22].

Purification of the extract of the Indonesian marine sponge of *Spongia* sp. led to the discovery of three new nitrogenous diterpenes, designated ceylonamides G–I (**53**–**55**), together with ceylonamide F (**12**). Among these marine natural products, **12** and **53** inhibited the proliferation of DU145 cells (IC_50_ = 18.8 and 6.9 µM, respectively) in a two-dimensional monolayer culture. Furthermore, these compounds were also effective on the spheroid of the three-dimensional DU145 cell culture model, with the minimum effective concentrations being 10 and 25 µM, respectively [23]. The search for the chemical composition of the Mexican sponge *Spongia tubulifera* resulted in the isolation and identification of two new spongian furanoditerpenes, 3β-hydroxyspongia-13(16),14-dien-2-one (**56**) and 19-dehydroxy-spongian diterpene 17 (**57**), along with four known terpenes, **58**–**61**. The absolute configurations of two new substances were determined through a comparison of the experimental ECD spectra with those calculated using time-dependent density functional theory (TDDFT). Weak cytotoxicities against the A549, A2058, HepG2, MCF7, and MiaPaca-2 cell lines (with IC_50_ values ranging from 91.3 to 11.7 µM) were observed for components **56**, **59**, and **61**. However, none of the metabolites showed any significant antibacterial activity against four bacteria *Acinetobacter baumannii*, *Pseudomonas aeruginosa*, *Klebsiella pneumoniae*, and *S. aureus* or potent antiviral activity against human HAdV5 and HAdV5-GFP adenoviruses [24]. A recent study indicated that diterpenes **56**–**60** displayed mitochondrial-mediated neuroprotective properties through direct interaction with cyclophilin D [25].

Dinorspongians A–F (**62**–**67**) and epoxynorspongians A–F (**68**–**73**) were found as twelve new metabolic compositions of a Chinese sponge *Spongia* sp. Structurally, compounds **62**–**67** were unprecedented dinorspongian diterpenes with a 3,4-seco-3,19-dinorspongian carbon framework, and **68**–**73** were the first reported 19-norspongian diterpenes with a 5,17-epoxy unit. The absolute configurations of these compounds were established through X-ray diffraction and quantum chemical calculation, respectively. The anti-proliferation properties of **62**–**73** against the HeLa, Huh7, RBL-2H3, and PC3 cell lines were evaluated. As a result, compound **72** showed moderate activity against the PC3 and PBL-2H3 cell lines (IC_50_ = 24.8 and 27.2 μM, respectively) [26]. A Red Sea *Spongia* sp. afforded a new 2,4-cyclized-3,4-secospongian diterpenoid, 17-dehydroxysponalactone (**74**), and a known norditerpene, 18-nor-3,17-dihydroxy-spongia-3,13(16),14-trien-2-one (**75**). Component **74** was found to be inactive in the cytotoxic bioassay against the P388, HuCCT, DLD-1, and CCD-966SK cell lines. However, it exhibited potent activity to inhibit superoxide anion (O_2_^−^) generation and elastase release (IC_50_ = 3.37 ± 0.21 and 4.07 ± 0.60 μM, respectively) [27].

A chemical investigation involving the aquaculture sponge *S. officinalis* yielded two new secondary metabolites 2β,3α,19-triacetoxy-17-hydroxyspongia-13(16),14-diene (**76**) and 18-nor-2,17-hydroxyspongia-1,4,13(16),14-quaien-3-one (**77**), together with six related known metabolites, **39**, **75**, and **78**–**81**. Quantum chemical calculations of NMR parameters and ECD were employed to determine the absolute configurations of two new isolates, **76** and **77**. All isolated compounds were evaluated for cytotoxicity against the K562, H69AR, ASPC-1, and MDA-MB-231 cell lines. Among them, constituents **75**, **76**, and **78** displayed cytotoxic activities against K562 (IC_50_ = 3.5, 7.3, and 6.4 μM, respectively), and **78** exhibited cytotoxicity against H69AR (IC_50_ = 9.5 μM). The signaling inhibition of HIF-1, Wnt, and STAT3/NF-κB and the activation of PPARγ/p53 of all isolated compounds were also evaluated. At the concentration of 20 μM, substances **75** and **78** displayed remarkable Wnt and HIF-1 inhibitory activity with inhibitory rates ranging from 88.5% to 84.7% [28]. The presence of an unreported bicyclic diterpenoid with an unprecedented penta-substituted carbon architecture, jellynolide A (**82**), was revealed in a further investigation involving the aquaculture sponge *S. officinalis*. Its absolute configuration was established through quantum chemical calculations of NMR parameters and ECD, too. Diterpene **82** was subjected to an array of bioassays including cytotoxicity against the K562, ASPC-1, MDA-MB-231, and H69AR cell lines, antipolymerase activities against COVID-19 RNA-dependent RNA polymerase, signaling inhibition of HIF-1, Wnt, STAT3/NF-κB, and activation of PPARγ/p53. As a result, **82** only showed obvious inhibition against HIF-1 and STAT3/NF-κB with inhibitory rates of 67.6% and 71.6% at 20 μM, respectively [29].

A new dinorspongian diterpene, dinorspongiapyridine (**83**), was reported as the chemical constituent of a South China Sea marine sponge *Spongia* sp. Metabolite **83** was the first member of the 3,4-seco-3,19-dinorspongian diterpene with a rare pyridyl D-ring system. Its structure along with absolute configuration was confirmed via X-ray diffraction. The anti-proliferative effects of **83** were evaluated against the K562, A549, Hela, HT29, SK-BR-3, and Huh7 cell lines. However, **83** showed no cytotoxicity against these cells at the concentration of 50 μmol/L [30].

In addition to **54**, 19,20-dihydroxyspongia-13(16),14-diene (**84**), 3β-acetoxyspongia-13(16),14-diene (**85**), and 3α-acetoxyspongia-13(16),14-diene (**86**) were identified as the chemical compositions of Red Sea marine sponge *Spongia irregularis*. The binding affinity of these compounds to different targets for HCV was examined via molecular docking experiments. However, the results revealed that none of them had obvious inhibitory potential [31]. A cemical investigation involving a sample of Red Sea sponge *Spongia* sp. led to the discovery of a new diterpene, spongianol (**87**), and a known one, 10-hydroxykahukuene B (**88**). The new compound **87** was a polyoxygenated and halogenated labdane diterpenoid, whose absolute configuration was established via the ECD calculation approach. The brominated diterpene **88** represented the first example of a natural product with a prenylated chamigrane skeleton that had been isolated from the sponge. In the cytotoxic bioassay, only compound **88** showed weak cytotoxicity against Huh7 cell lines (inhibitory rate of 17% at 50 µM). However, both terpenes were inactive in the screening experiments of antibacterial activity against *S. aureus* and anti-inflammatory activity [15].

Another study on a Red Sea sponge *Spongia* sp. showed the presence of three new diterpenes named spongenolactones A–C (**89**–**91**). Compounds **89**–**91** were the first reported 5,5,6,6,5-pentacyclic spongian diterpenes with an β-hydroxy group at C-1. The anti-proliferative effects of **89**–**91** against the Huh7 cell line were evaluated. However, none of them showed notable cytotoxic activity against this cancer cell line, whereas **89** and **90** displayed weak antibacterial properties against *S. aureus* and weak inhibition of O_2_^−^ generation in the corresponding bioassays [32]. Further chemical investigation of this sponge revealed the discovery of four new 3,4-seco-3,19-dinorspongian lactones, secodinorspongins A–D (**92**–**95**), along with a spongian lactone, sponginolide (**96**). The absolute configurations of compounds **92** and **94**–**96** were established through a comparison of the experimental and calculated ECD spectra. Compound **92** exhibited antibacterial activity against *S. aureus* and compounds **95** and **96** displayed anti-inflammatory effect in the suppression of O_2_^−^ [33].

## 4. Sesterterpenes

**Figure 3:**
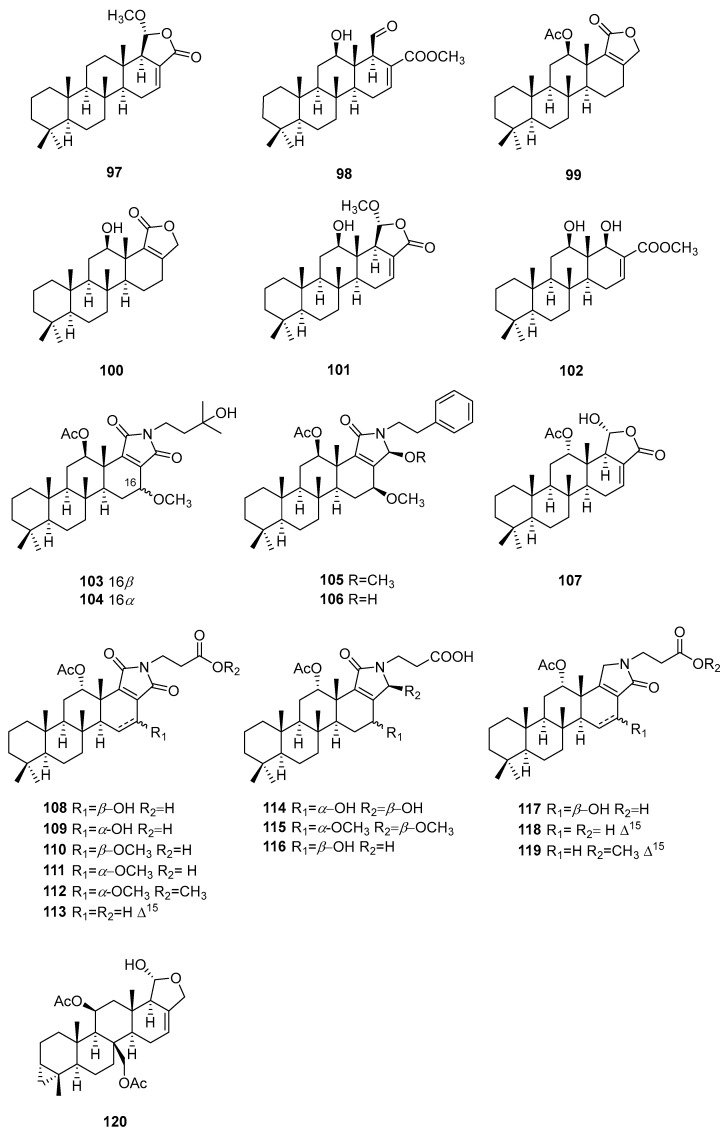
Sesterterpenes obtained from the genus *Spongia*.

All of the sesterterpenes of the genus *Spongia* possess a carbon skeleton named scalarane, which is a tetracyclic carbon architecture of four fused six-membered rings (Figure 3). In some cases, sesterterpenoids have ring E and form a pentacyclic system.

Two new scalarane-type sesterterpenes **97** and **98** were identified as chemical compositions of the Korean marine sponge *Spongia* sp. They were tested for their farnesoid X-activated receptor (FXR) antagonistic and cytotoxic activities. However, neither of them were active [34]. For the Bornean sponge *Spongia* sp., scalarolide acetate (**99**), scalarolide (**100**), 12-*O*-deacetyl-12-*epi*-19-*O*-methylscalarin (**101**), and methyl 18-hydroxy-19-norscalar-16-en-20-carboxylate (**102**) were reported as its metabolic components. Metabolites **99**–**101** exhibited strong cytotoxic activities against adult T-cell leukemia, S1T cells [35].

A further study on the chemical compositions of Korean marine sponge *Spongia* sp. resulted in the discovery of four new scalarane sesterterpenes named scalalactams A–D (**103**–**106**). These terpenes were characterized by an isopentanyl alcohol or a phenylethyl group substituted at the lactam ring. All of them were inactive in the FXR antagonistic and cytotoxic bioassays [36]. The Mexican marine sponge *S. tubulifera* afforded scalarin (**107**). This substance was evaluated for cytotoxic activity against the A549, A2058, HepG2, MCF7, and MiaPaca-2 cell lines, antibacterial activity against *A. baumannii*, *P. aeruginosa*, *K. pneumoniae*, and *S. aureus*, and antiviral activity against human HAdV5 and HAdV5-GFP adenoviruses. However, no obvious bioactivity was observed in these bioassays [24].

Twelve unreported scalaranes, namely scalimides A–L (**108**–**119**), were found in the Philippine marine sponge *Spongia* sp. These constituents furnished a lactam ring with different degrees of oxidation and substitutions. All of them were evaluated for antibacterial activity against the Gram-positive bacteria *Micrococcus luteus* and *S. aureus* and *Bacillus subtilis* and the Gram-negative bacteria *Salmonella typhimurium*, *K. pneumoniae*, and *Escherichia coli*. As a result, only compound **117** was active against all bacteria, with MIC values ranging from 4 to 64 μg/mL. Moreover, components **108**–**119** exhibited moderate anticancer effects against MCF7, with EC_50_ values ranging from 13.7 to 73.9 μM [37].

12-Deacetoxy-4-demethyl-11,24-diacetoxy-3,4-methylenedeoxoscalarin (**120**) was a new sesterterpene yielded by the Indonesian marine sponge *Spongia* cf. *agaricina*. This substance featured the fusion of a cyclopropane ring and ring A and displayed antibacterial activity against *Arthrobacter crystallopoietes* and *Bacillus megaterium* [38].

## 5. Meroterpenes

**Figure 4:**
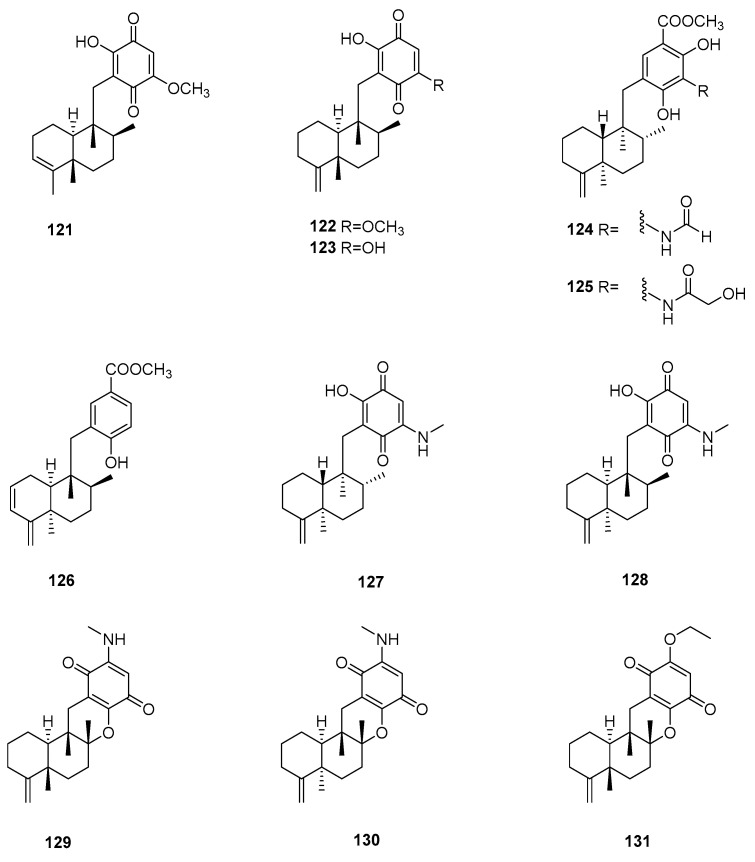

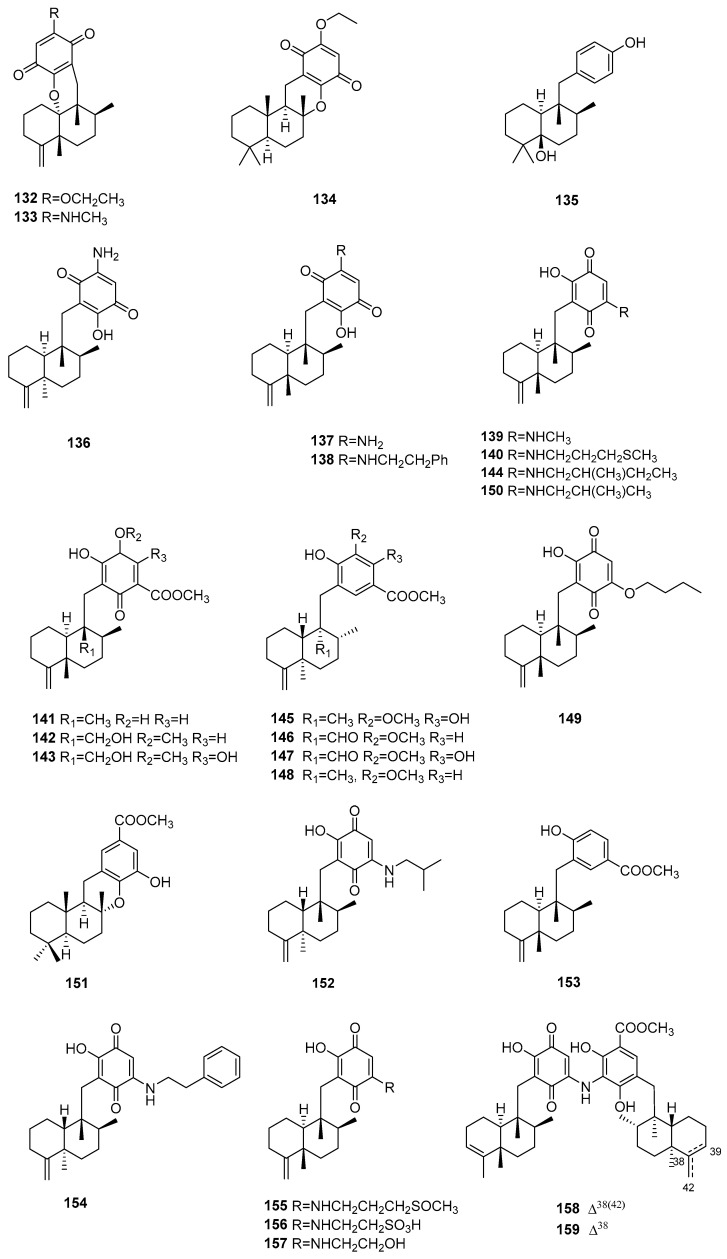

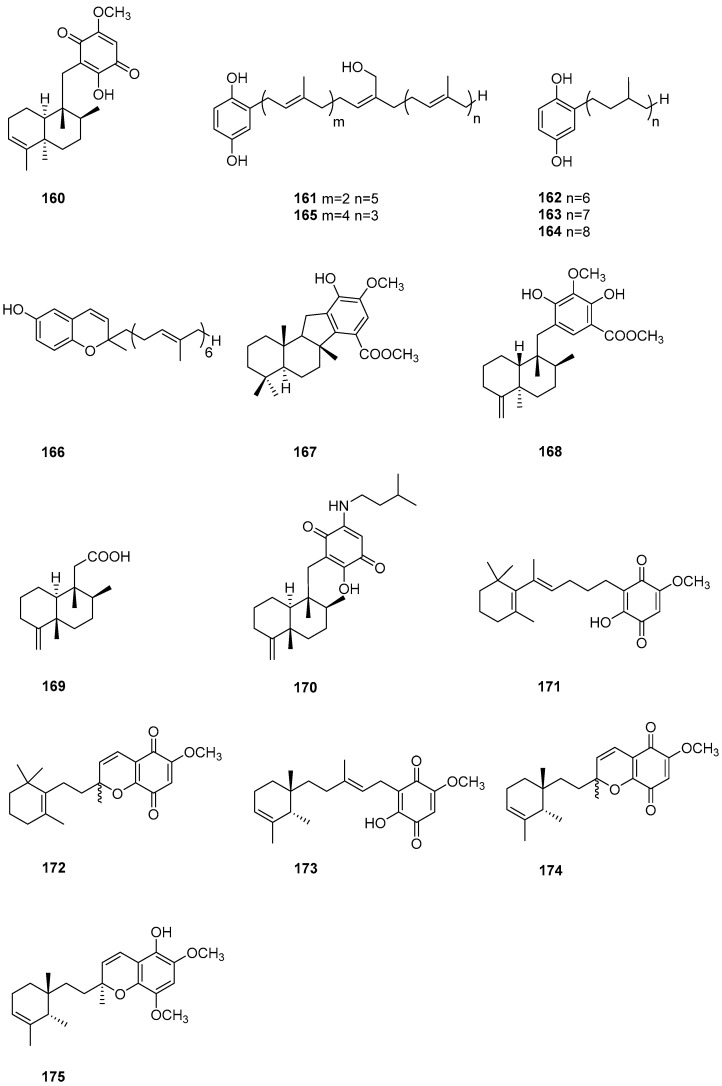
Meroterpenes obtained from the genus *Spongia*.

This category of terpenes are the hybrids of sesquiterpenes and quinones or phenols, except for smenospongic acid, which is a degradation product (Figure 4).

Three meroterpenes, isospongiaquinone (**121**), ilimaquinone (**122**), and smenoquinone (**123**), were isolated from the South China Sea marine sponge *Spongia* sp. Components **121** and **122** exhibited phytoregulating activity for agricultural plants [39]. An investigation involving the South China Sea marine sponge *Spongia pertusa* led to the discovery of nine new secondary metabolites, **124**–**130**, **133**, and **135**, three artifacts, **131**, **132**, and **134**, and three known compounds, **136**–**138**. Their absolute configurations were established through comparisons of experimental and calculated ECD spectra. All of these components were evaluated for cytotoxicities against the U937, A549, HeLa, and HepG2 cell lines. The results showed that metabolites **136**–**138** exhibited the most potent cytotoxic activities against U937 (IC_50_ = 2.8, 1.5, and 0.6 μM, respectively). Additionally, **129** exhibited CDK-2 affinity (*K*_d_ = 4.8 μM) [40]. Further in-depth study disclosed that smenospongine (**137**) preferentially eliminates breast cancer stem-like cells via p38/AMPKα pathways [41].

Langcoquinones A (**139**) and B (**140**), along with four known meroterpenoids, **141**–**144**, in addition to the previously reported compounds **122**, **137**, and **138**, were identified as the chemical compositions of the Vietnamese marine sponge *Spongia* sp. These components were assessed for antibacterial activities against *B. subtilis*, *S. aureus*, *K. pneumoniae*, and *E. coli*. As a result, **122**, **141**, **137**, **138**, and **144** displayed selective antibacterial activities against *B. subtilis* and *S. aureus* (MIC = 6.25–12.5 μM). As observed, the hydroxy group at C-14 led to the loss of antibacterial activity against the Gram-positive bacteria [42]. Four new meroterpenes, namely langconols A–C (**145**–**147**) and langcoquinone C (**148**), as well as two known ones, **149** and **150**, were identified in further studies on the sponge *Spongia* sp. The antibacterial assays indicated that **148** and **150** exhibited remarkable activity against *B. subtilis* and *S. aureus* (MIC = 6.25–25.0 μM, respectively), while **145** and **147** displayed strong antibacterial activities against *B. subtilis* (MICs = 12.5 and 25.0 μM, respectively). Moreover, isolates **122**, **137**–**140**, **134**, **147**, **148**, and **150** displayed significant cytotoxic activities against the HeLa, MCF7, A549, and WI-38 cell lines (IC_50_ = 3.0–9.7 μM) [43].

The Xisha marine sponge *Spongia* sp. afforded five meroterpenes, **150**–**154**, in addition to the previously reported **141**. Compounds **150**, **152**, and **154** showed antifungal activities against *Trichophyton rubrum*, *Trichophyton mentaqrophytes*, and *Candida albicans* (MIC = 12.5–25 μg/mL) [44]. The Vietnamese sponge *Spongia* sp. yielded three metabolites, langcoquinones D–F (**155**–**157**). Component **155** showed antibacterial effects against *B. subtilis* and *S. aureus* (MIC = 12.5 and 25.0 μM, respectively) and cytotoxic properties toward the HeLa, MCF7, A549, and WI-38 cell lines (IC_50_ = 8.6, 5.9, 8.9, and 5.6 μM, respectively) [45]. An investigation involving the Red Sea marine sponge *S. irregularis* disclosed the presence of four meroterpenes, **158**–**161**. Molecular docking calculations inferred the HCV inhibitory potential of **159** [31]. A study on the Aegean marine sponge *Spongia* sp. revealed the existence of five substances, **162**–**166**. Compound **163** exhibited the best cytotoxic activity toward AGS cells (IC_50_ = 0.99 μM) [46].

Purification of the extract of South China Sea marine sponge *Spongia pertusa* yielded two new merosesquiterpenoids, **167** and **168**, together with nine known related analogs, **124**, **125**, **138**, **144**, **150**, **152**, **155**, **169**, and **170**. The moderate antifungal activity of components **144**, **150**, **152**, **155**, and **170** against *T. rubrum*, *T. mentaqrophytes*, and *C*. *albicans* (MIC_50_ = 12.5–100 μg/mL) was observed [47]. Bioassay-guided fractionation of the extract of Japanese marine sponge *Spongia* (*Heterofibria*) sp. afforded two new sesquiterpene quinones, namely metachromins X (**171**) and Y (**172**), together with three known analogs, **173**–**175**. The structure of **171** was confirmed through total synthesis. The absolute configuration of **172** was determined through a combination of chiral HPLC and CD spectroscopy. All of these chemical constituents showed weak cytotoxicity against HeLa cells with (IC_50_ = 64, 76, 53, 73, and 89 μM, respectively). Additionally, **171** and **173** displayed arrest effects on the cell cycle progression of HeLa/Fucci2 cells at the S/G2/M phase [48].

## 6. Linear Furanoterpenoids

**Figure 5:**
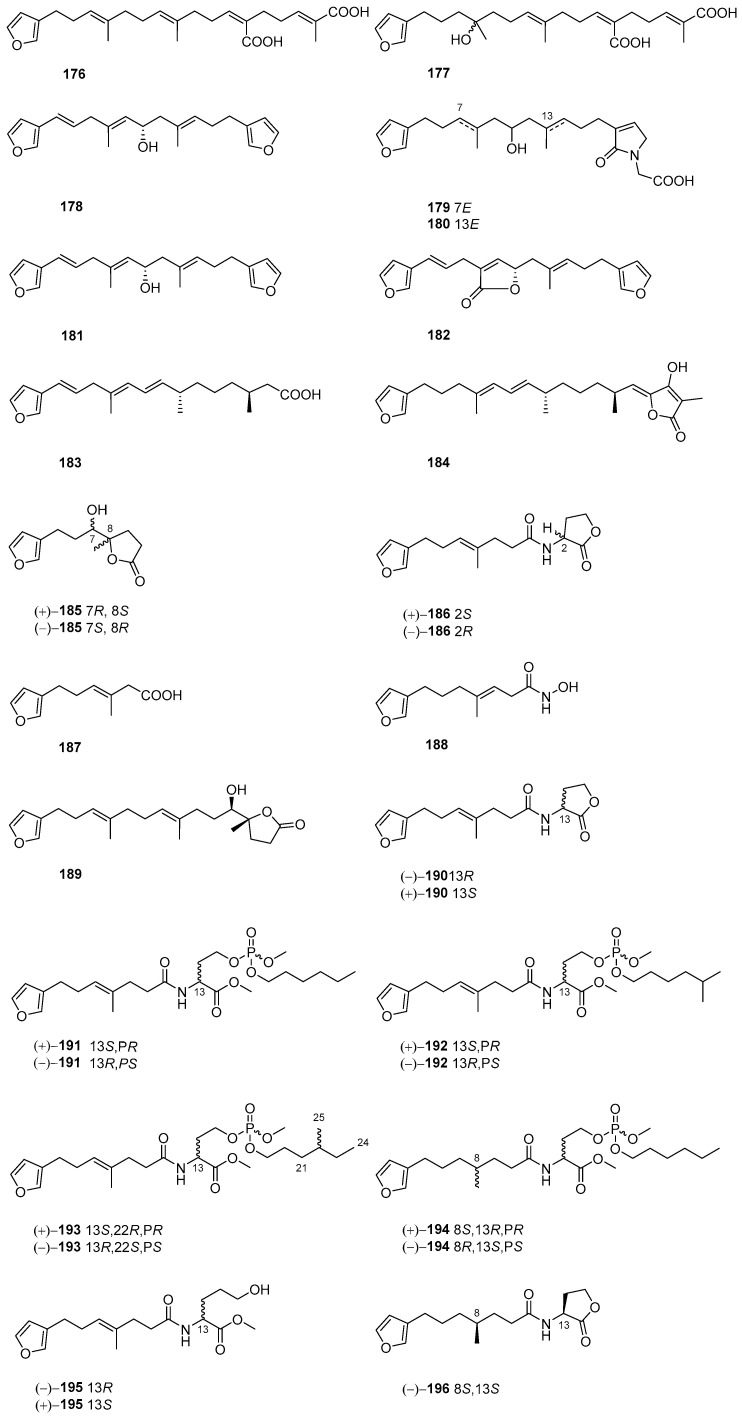
Linear furanoterpenoids obtained from the genus *Spongia*.

This group of terpenes do not possess a cyclic carbon ring but are composed of a furan ring. In some compounds, an additional γ-lactone or γ-lactam ring is present (Figure 5).

Demethylfurospongin-4 (**176**), furofficin (**177**), furospongin-1 (**178**), and spongialactams A (**179**) and B (**180**) have been reported as metabolic components of the Mediterranean marine sponge *S. officinalis*, which was recognized through untargeted LC–MS metabolomics and molecular networking [49]. Compound **178** was also found in the Indonesian marine sponge *Spongia* sp. This chemical constituent displayed PTP1B, TCPTP, and VHR inhibitory activities (IC_50_ = 9.9, 9.6 and 11 μM, respectively) [50]. The marine sponge *S. officinalis* collected off of Weizhou Island was found to be a source of furanoterpenes **181**–**184** [51]. Further study on this species resulted in the discovery of two pairs of racemic furan butanolides, (±)-sponalisolides A (**185**) and B (**186**), which were characterized by an unprecedented furan-bearing alkyl chain connecting either a butanolide or an *N*-acyl homoserine lactone moiety. Biomimetic total synthesis was conducted to confirm their structures as well as absolute configurations unambiguously. All of the new compounds displayed quorum-sensing inhibitory activity against *Pseudomonas aeruginosa* [52].

The Red Sea marine sponge *Spongia* sp. Afforded three new compounds, **187**–**189**, and one known analogue, **190**. These isolates did not exhibit cytotoxic against the P388, HuCCT DLD-1, and CCD-966SK cell lines. However, compound **190** was found to display significant inhibitory activity against O_2_^−^ generation (IC_50_ = 5.31 ± 1.52 μM) [27]. The cultured sponge *S. officinalis* yielded six new chemical constituents, (±)-**191**-(±)-**195** and (−)-**196**, as well as two previously reported compounds, (−)-**187** and (±)-**190**. Notably, metabolites (±)-**191**-(±)-**195** were four pairs of rare phosphate triesters. The absolute configurations of new isolates, **191**–**195**, were determined through quantum chemical calculations of NMR parameters and ECD. Moderate cytotoxicities against the MDA-MB-231, K562, and ASPC-1 cell lines (IC_50_ = 17.4, 28.0, and 11.0 μM, respectively) were observed for (+)-**191**. Moreover, (±)-**192** displayed remarkable Wnt and HIF1 signaling inhibition [29].

## 7. Steroids

**Figure 6:**
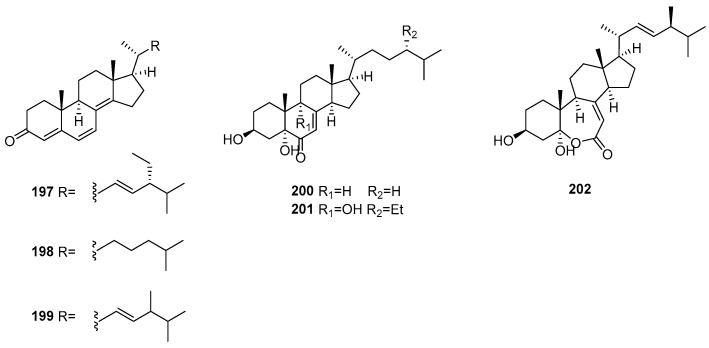
Steroids obtained from the genus *Spongia*.

The Xisha sponge *Spongia* sp. afforded three unsaturated steroids, **197**–**199** (Figure 6). Among them, compound **199** exhibited antifungal activity against *T. rubrum* (MIC = 25 μg/mL) [44]. The Red Sea sponge *Spongia* sp. yielded another unsaturated sterol, **200**. This chemical constituent was evaluated for cytotoxicity against the P388, DLD-1, and CCD-966SK cell lines and the inhibition of O_2_^−^ generation and elastase release. However, it displayed no obvious activity in the bioassays [27]. In a continuous study, two polyoxygenated steroids, **201** and **202**, were identified. Intriguingly, **202** is the fourth steroid that possesses a rare seven-membered lactone B ring. The assignment of the absolute configuration of C-24 of the side chain in **201** was achieved based on the chemical shift differences of C-26 and C-27, which was further verified through a comparison of experimental and calculated ECD spectra. Neither of these two substances exhibited cytotoxicity against the Huh7 cell line or antibacterial activity against *S. aureus* or anti-inflammatory activity [15].

## 8. Alkaloids

This category of compounds is composed of true alkaloids and pseudoalkaloids. Biogenetically, the former are derived directly from amino acids, and the latter are derived from purine or other non-amino acids.

**Figure 7:**
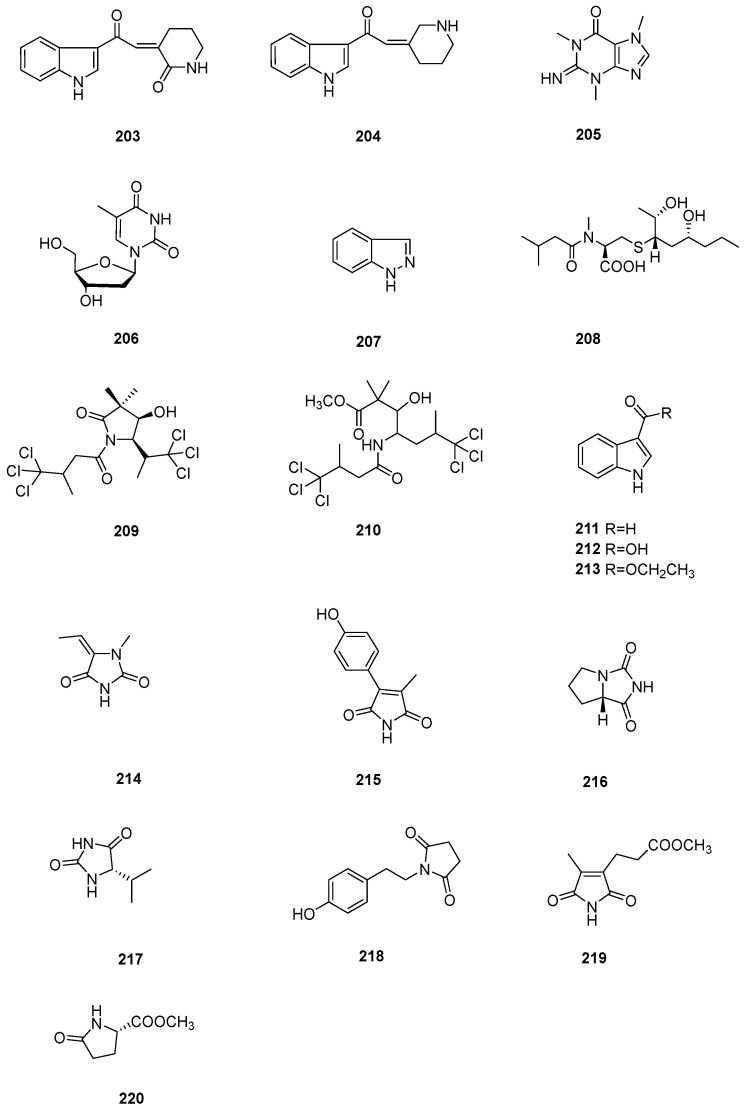
Alkaloids obtained from the genus *Spongia*.

Two indole alkaloids, **203** and **204** (Figure 7), were isolated from the Korean marine sponge *Spongia* sp. They did not display obvious FXR inhibitory activity or cytotoxicity against CV-1 cells [53]. Compounds **205**–**208** were reported to be metabolic components of the Red Sea sponge *S. irregularis*. Docking studies of these metabolites were carried out to evaluate their binding affinity to different targets for HCV. However, the results revealed that none of them had obvious inhibitory potential [31]. The Red Sea sponge *Spongia* sp. afforded amides **209** and **210**, in addition to the previously reported amide **206**. Chemical constituent **206** exhibited weak antibacterial activity against *S. aureus*, and chemical constituent **210** displayed weak cytotoxicity against Huh7 cells. Moreover, components **209** and **210** showed moderate elastase release inhibition (IC_50_ = 17.23 ± 2.45 and 14.60 ± 2.24 µM, respectively) [15].

The South China Sea marine sponge *Spongia* sp. is a source of alkaloids **207** and **211**–**213** [30]. Further study on this sample resulted in the discovery of two new alkaloids, spongimides A (**214**) and B (**215**), along with five known ones, **216**–**220**. X-ray diffraction experiments confirmed the structures of two new compounds. Interestingly, **214**, **215**, and **217** are the first examples of 2,4-imidazolidinedione found in this genus. These two new chemical constituents did not exhibit cytotoxicity against the K562 cell line or antibacterial activities against *Salmonella paratyphi* and *E. coli* [54].

## 9. Other Miscellaneous Substances

**Figure 8:**
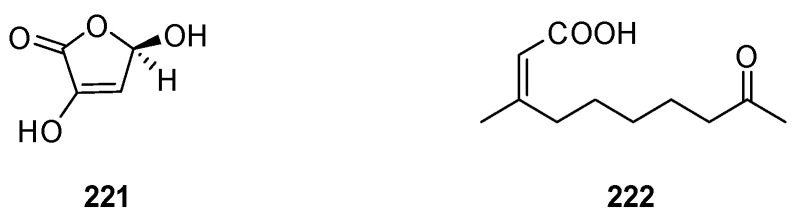
Other miscellaneous compounds obtained from the genus *Spongia*.

Chemical investigation of the Red Sea sponge *S. irregularis* afforded 3,5-dihydroxyfuran-2(5*H*)-one (**221**) (Figure 8). Docking experiments revealed that this compound does not show obvious inhibitory effects against different targets of HCV [31]. A new α,β-unsaturated fatty acid, (*Z*)-3-methyl-9-oxodec-2-enoic acid (**222**), was found in the Red Sea sponge *Spongia* sp. This compound was subjected to several bioassays including cytotoxicity against the Huh7 cell line, antibacterial activity against *S. aureus*, and anti-inflammatory activity. However, it did not show any obvious activity [15].

## 10. Discussion

**Figure 9:**
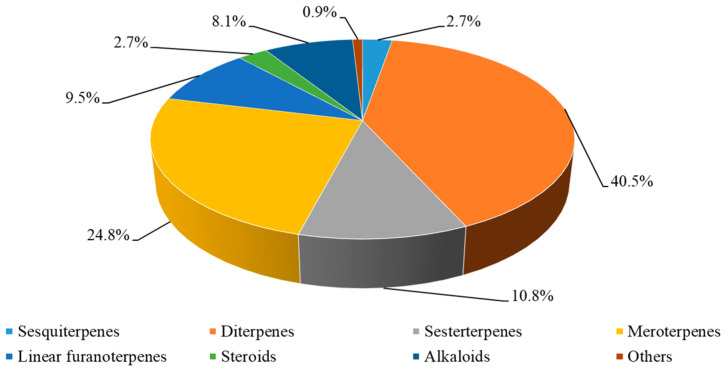
Chemical profile of secondary metabolites from the genus *Spongia*.

A total of 222 compounds from a variety of species of genus *Spongia* were reported from mid-2015 to the beginning of 2024, covering a period of nine years (Table S1). The substances illustrated in this work could be categorized as sesquiterpenes, diterpenes, sesterterpenes, meroterpenes, linear furanoterpenes, steroids, alkaloids, and other miscellaneous substances (Figure 9, Table S1). Most of these metabolites could be grouped into two major chemical classes: diterpenes and meroterpenes. Among them, diterpenes were the predominant chemical constituents, accounting for 40.5%, almost half of the total. These diterpenes were distinguished by their unique structural features, such as fused ring systems, stereochemical complexity, and functional group diversity.

**Figure 10:**
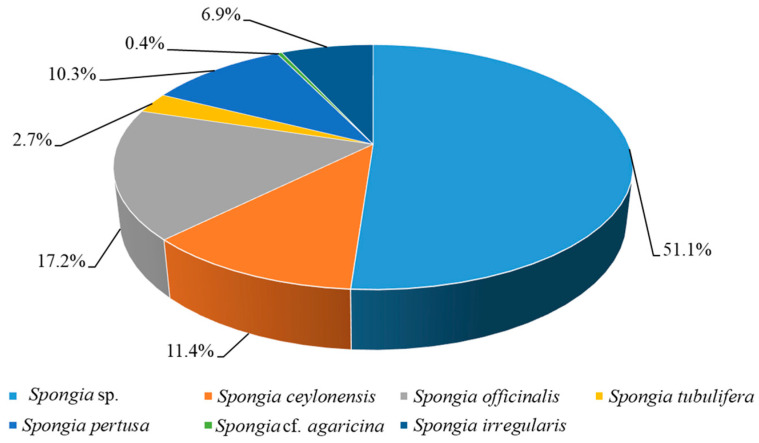
Reported compounds from different species of the genus *Spongia*.

Chemical investigations have been conducted on the species *Spongia ceylonensis*, *Spongia officinalis*, *Spongia tubulifera*, *Spongia pertusa*, *Spongia* cf. *agaricina*, *Spongia irregularis*, and unclearly identified *Spongia* sp. (Figure 10, Table S1). In terms of the number of isolated substances, except the unclearly identified *Spongia* sp., *S. officinalis* was the most prolific species, yielding 45 compounds. (Notably, some compounds were obtained in different species and were counted separately in each species).

**Figure 11:**
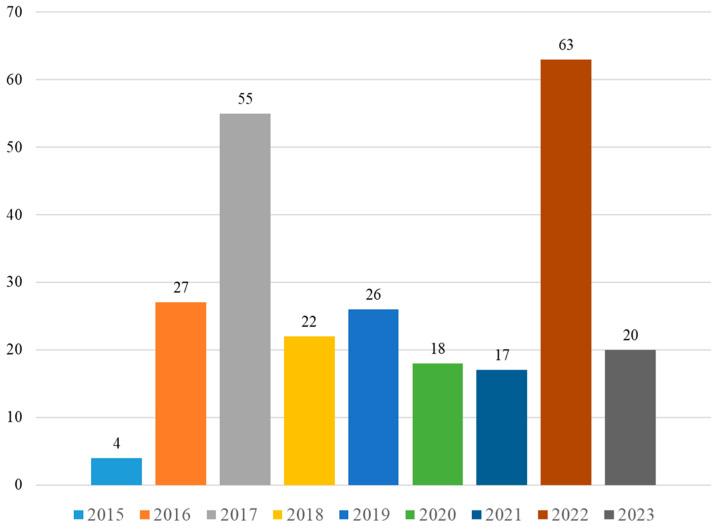
Annually reported compounds from the genus *Spongia*.

As outlined in Figure 11 and Table S1, 222 metabolites were obtained from different species of the genus *Spongia* from mid-2015 to the beginning of 2024. This indicated that more than 20 secondary metabolites were afforded by the genus *Spongia* every year. Among these years, most compounds were found in the *Spongia* sponges in 2022. (Notably, some compounds were obtained in different years and were counted separately in each year).

## 11. Conclusions

As reported, some secondary metabolites, such as linear furanoterpenoids (±)-sponalisolides A (**185**) and B (**186**) [52], display fascinating structures, which makes them attractive targets for synthetic chemists. Moreover, some substances, for instance, spongian diterpenes **78** and **79** [28], exhibit significant activity including cytotoxic, anti-inflammatory, antibacterial, neuroprotective, and PTP1B inhibitory activities, which are worth further in-depth study by pharmacologists. So many compounds with distinct structural features and biological properties were reported in such a short period, indicating that *Spongia* sponges are unabated sources of novel secondary metabolites.

Due to the low yields of some important natural products from *Spongia* sponges, the exploration of silencing gene expression and large-scale fermentation for industry will likely be research hotspots in the future.

## Data Availability

Not applicable.

## Supplementary Materials

The following supporting information can be downloaded at: https://www.mdpi.com/article/10.3390/md22050213/s1. Table S1: Secondary metabolites of the genus *Spongia* from mid-2015 to the beginning of 2024.
