# Supplementary material for: Spongia Sponges: Unabated Sources of Novel Secondary Metabolites
Source: Mar Drugs. 2024 May 7;22(5):213. doi: 10.3390/md22050213 (PMC11123444; doi:10.3390/md22050213)
Supplement: Supplementary file 1 [file marinedrugs-22-00213-s001.zip › marinedrugs-2967897-supplementary.pdf]

Supplementary Materials

Table S1. Secondary metabolites of the genus *Spongia* from mid-2015 to the beginning of 2024.

No	Name	Class	Species	Locality	Bioassays	Ref
**1**	(+/-)-spongiterpene	sesquiterpene	*Spongia* sp.	Zhanjiang, Guangdong, China	—[1]	[14]
**2**	dehydroloIiolide	sesquiterpene	*Spongia* sp.	Zhanjiang, Guangdong, China	—	[14]
**3**	loliolide	sesquiterpene	*Spongia* sp.	Zhanjiang, Guangdong, China	no cytotoxicity against K562, A549, Hela, HT29, SK-BR-3, Huh7, RBL-2H3 and PC3 cell lines (MTT asssay, triplicate)	[14]
**4**	dehydrovomifoliol	sesquiterpene	*Spongia* sp.	Zhanjiang, Guangdong, China	—	[14]
**5**	3β-hydroxy-5α,6α-epoxy-7-megastimen-9-one	sesquiterpene	*Spongia* sp.	Zhanjiang, Guangdong, China	—	[14]
**6**	pacifenol	sesquiterpene	*Spongia* sp.	Jeddah, Saudi Arabia	no cytotoxicity against Huh7 cell line (MTT asssay, triplicate); no antibacterial activity against *Staphylococcus aureus*; no anti-inflammatory activity toward inhibiting superoxide anion (O_2^-) generation and elastase release (IC_{50} > 20 μM)	[15]
**7**	ceylonamide A	diterpene	*Spongia ceylonensis*	Tiwoho, North Sulawesi, Indonesia	inhibitory against RANKL-induced osteoclastogenesis (IC_{50} = 13 μM)	[17]
					no inhibitory against ubiquitin-specific protease 7 (USP7) (IC_{50} > 50 μM)	[19]
**8**	ceylonamide B	diterpene	*S. ceylonensis*	Tiwoho, North Sulawesi, Indonesia	inhibitory against RANKL-induced osteoclastogenesis (IC_{50} = 18 μM)	[17]
					no inhibitory against USP7 (IC_{50} > 50 μM)	[19]
**9**	ceylonamide C	diterpene	*S. ceylonensis*	Tiwoho, North Sulawesi, Indonesia	no inhibitory against RANKL-induced osteoclastogenesis (IC_{50} > 50 μM)	[17]
					no inhibitory against USP7 (IC_{50} > 50 μM)	[19]
**10**	ceylonamide D	diterpene	*S. ceylonensis*	Tiwoho, North Sulawesi, Indonesia	no inhibitory against RANKL-induced osteoclastogenesis (IC_{50} > 50 μM)	[17]
					no inhibitory against USP7 (IC_{50} > 50 μM)	[19]
**11**	ceylonamide E	diterpene	*S. ceylonensis*	Tiwoho, North Sulawesi, Indonesia	no inhibitory against RANKL-induced osteoclastogenesis (IC_{50} > 50 μM)	[17]
					no inhibitory against USP7 (IC_{50} > 50 μM)	[19]
**12**	ceylonamide F	diterpene	*S. ceylonensis*	Tiwoho, North Sulawesi, Indonesia	no inhibitory against RANKL-induced osteoclastogenesis (IC_{50} > 50 μM)	[17]

No	Name	Class	Species	Locality	Bioassays	Ref
					no inhibitory against USP7 (IC_{50} > 50 μM)	[19]
			Spongia sp.	Biak, Indonesia	cytotoxicity against DU145 cell line (IC_{50} = 18.8 μM)	[23]
**13**	15α,16-dimethoxyspongi-13-en-19-oic acid	diterpene	*S. ceylonensis*	Tiwoho, North Sulawesi, Indonesia	no inhibitory against RANKL-induced osteoclastogenesis (IC_{50} > 50 μM)	[17]
**14**	haumanamide	diterpene	*S. ceylonensis*	Tiwoho, North Sulawesi, Indonesia	no inhibitory against RANKL-induced osteoclastogenesis (IC_{50} > 50 μM)	[17]
**15**	15-oxospongi-13-en-19-oic acid	diterpene	*S. ceylonensis*	Tiwoho, North Sulawesi, Indonesia	no inhibitory against RANKL-induced osteoclastogenesis (IC_{50} > 50 μM)	[17]
					no inhibitory against USP7 (IC_{50} > 50 μM)	[19]
**16**	16-oxospongi-13-en-19-oic acid	diterpene	*S. ceylonensis*	Tiwoho, North Sulawesi, Indonesia	no inhibitory against RANKL-induced osteoclastogenesis (IC_{50} > 50 μM)	[17]
					no inhibitory against USP7 (IC_{50} > 50 μM)	[19]
**17**	spongiabutenolide A	diterpene	*S. ceylonensis*	Tiwoho, North Sulawesi, Indonesia	no inhibitory against RANKL-induced osteoclastogenesis (IC_{50} > 50 μM)	[17]
					no inhibitory against USP7 (IC_{50} > 50 μM)	[19]
**18**	spongiabutenolide B	diterpene	*S. ceylonensis*	Tiwoho, North Sulawesi, Indonesia	no inhibitory against RANKL-induced osteoclastogenesis (IC_{50} > 50 μM)	[17]
					no inhibitory against USP7 (IC_{50} > 50 μM)	[19]
**19**	spongia-13(16),14-dien-19-oic acid	diterpene	*S. ceylonensis*	Tiwoho, North Sulawesi, Indonesia	no inhibitory against RANKL-induced osteoclastogenesis (IC_{50} > 50 μM)	[17] [18]
					no inhibitory against USP7 (IC_{50} > 50 μM)	[19]
**20**	spongiadiol	diterpene	*S. ceylonensis*	Tiwoho, North Sulawesi, Indonesia	no inhibitory against RANKL-induced osteoclastogenesis (IC_{50} > 50 μM)	[17]
**21**	isospongiadiol	diterpene	*S. ceylonensis*	Tiwoho, North Sulawesi, Indonesia	no inhibitory against RANKL-induced osteoclastogenesis (IC_{50} > 50 μM)	[17]
**22**	ceylonin A	diterpene	*S. ceylonensis*	Tiwoho, North Sulawesi, Indonesia	inhibitory against RANKL-induced osteo-clastogenesis (inhibiinhibition rates 70%)	[18]
					no inhibitory against USP7 (IC_{50} > 50 μM)	[19]
**23**	ceylonin B	diterpene	*S. ceylonensis*	Tiwoho, North Sulawesi, Indonesia	no inhibitory against RANKL-induced osteoclastogenesis (IC_{50} > 50 μM)	[18]
					no inhibitory against USP7 (IC_{50} > 50 μM)	[19]
**24**	ceylonin C	diterpene	*S. ceylonensis*	Tiwoho, North Sulawesi, Indonesia	no inhibitory against RANKL-induced osteoclastogenesis (IC_{50} > 50 μM)	[18]
					no inhibitory against USP7 (IC_{50} > 50 μM)	[19]
**25**	ceylonin D	diterpene	*S. ceylonensis*	Tiwoho, North Sulawesi, Indonesia	inhibitory against RANKL-induced osteo-	[18]

No	Name	Class	Species	Locality	Bioassays	Ref
					clastogenesis (inhibiinhibition rates 28%)	
					no inhibitory against USP7 (IC_{50} > 50 μM)	[19]
**26**	ceylonin E	diterpene	*S. ceylonensis*	Tiwoho, North Sulawesi, Indonesia	inhibitory against RANKL-induced osteo-clastogenesis (inhibiinhibition rates 47%)	[18]
					no inhibitory against USP7 (IC_{50} > 50 μM)	[19]
**27**	ceylonin F	diterpene	*S. ceylonensis*	Tiwoho, North Sulawesi, Indonesia	inhibitory against RANKL-induced osteo-clastogenesis (inhibiinhibition rates 31%)	[18]
					no inhibitory against USP7 (IC_{50} > 50 μM)	[19]
**28**	ceylonin G	diterpene	*S. ceylonensis*	Tiwoho, North Sulawesi, Indonesia	no inhibitory against USP7 (IC_{50} > 50 μM)	[19]
**29**	ceylonin H	diterpene	*S. ceylonensis*	Tiwoho, North Sulawesi, Indonesia	no inhibitory against USP7 (IC_{50} > 50 μM)	[19]
**30**	ceylonin I	diterpene	*S. ceylonensis*	Tiwoho, North Sulawesi, Indonesia	no inhibitory against USP7 (IC_{50} > 50 μM)	[19]
**31**	*ent*-13-norisocopalen-15-al-18-oic acid	diterpene	*S. ceylonensis*	Tiwoho, North Sulawesi, Indonesia	inhibitory against USP7 (IC_{50} = 8.2 μM)	[19]
**32**	3-nor-spongiolide A	diterpene	*Spongia officinalis*	Weizhou Island, Guangxi, China	no cytotoxicity against HL-60 cell line (MTT asssay, triplicate)	[20]
**33**	spongiolide A	diterpene	*S. officinalis*	Weizhou Island, Guangxi, China	no cytotoxicity against HL-60 cell line (MTT asssay, triplicate)	[20]
**34**	spongiolide B	diterpene	*S. officinalis*	Weizhou Island, Guangxi, China	no cytotoxicity against HL-60 cell line (MTT asssay, triplicate)	[20]
**35**	spongia-13(16),14-diene	diterpene	*S. officinalis*	Weizhou Island, Guangxi, China	no cytotoxicity against HL-60 cell line (MTT asssay, triplicate)	[20]
**36**	18-nor-3,17-dihydroxyspongia-3,13 (16),14-trien-2-one	diterpene	*S. officinalis*	Weizhou Island, Guangxi, China	no cytotoxicity against HL-60 cell line (MTT asssay, triplicate)	[20]
**37**	spongiatriol	diterpene	*S. officinalis*	Weizhou Island, Guangxi, China	no cytotoxicity against HL-60 cell line (MTT asssay, triplicate)	[20]
				Ximao Island, Hainan, China	—	[22]
**38**	epispongiatriol	diterpene	*S. officinalis*	Weizhou Island, Guangxi, China	no cytotoxicity against HL-60 cell line (MTT asssay, triplicate)	[20]
				Ximao Island, Hainan, China	anti-inflammatory activity toward inhibiting lipopolysaccharide (LPS)-induced nitric oxide (NO) production (IC_{50} = 20 ± 2 μM)	[22]
**39**	isospongiatriol	diterpene	*S. officinalis*	Weizhou Island, Guangxi, China	no cytotoxicity against HL-60 cell line (MTT asssay, triplicate)	[20]
				Ximao Island, Hainan, China	no anti-inflammatory activity toward inhibiting LPS-induced NO production (IC_{50} >60 μM)	[22]

No	Name	Class	Species	Locality	Bioassays	Ref
				Shui Niupo Village, Danzhou City, Hainan Province, China	no cytotoxicity against K562, H69AR, ASPC-1 and MDA-MB-231 cell lines (SRB assay and MTT assay, triplicate); no signalling inhibition of HIF-1, Wnt and STAT3/NF-κB, or activation of PPARγ and p53	[28]
**40**	17,19-dihydroxyspongia-13(16),14-dien-2,3-dione	diterpene	*S. officinalis*	Weizhou Island, Guangxi, China	no cytotoxicity against HL-60 cell line (MTT asssay, triplicate)	[20]
**41**	spongiain A	diterpene	*Spongia* sp.	Zhanjiang, Guangdong, China	no cytotoxicity against K562, A549, Hela, HT29, SK-BR-3, Huh7, RBL-2H3 and PC3 cell lines (MTT asssay, triplicate)	[21]
**42**	spongiain B	diterpene	*Spongia* sp.	Zhanjiang, Guangdong, China	no cytotoxicity against K562, A549, Hela, HT29, SK-BR-3, Huh7, RBL-2H3 and PC3 cell lines (MTT asssay, triplicate)	[21]
**43**	spongiain C	diterpene	*Spongia* sp.	Zhanjiang, Guangdong, China	no cytotoxicity against K562, A549, Hela, HT29, SK-BR-3, Huh7, RBL-2H3 and PC3 cell lines (MTT asssay, triplicate)	[21]
**44**	spongiain D	diterpene	*Spongia* sp.	Zhanjiang, Guangdong, China	no cytotoxicity against K562, A549, Hela, HT29, SK-BR-3, Huh7, RBL-2H3 and PC3 cell lines (MTT asssay, triplicate)	[21]
**45**	spongiain E	diterpene	*Spongia* sp.	Zhanjiang, Guangdong, China	no cytotoxicity against K562, A549, Hela, HT29, SK-BR-3, Huh7, RBL-2H3 and PC3 cell lines (MTT asssay, triplicate)	[21]
**46**	spongiain F	diterpene	*Spongia* sp.	Zhanjiang, Guangdong, China	no cytotoxicity against K562, A549, Hela, HT29, SK-BR-3, Huh7, RBL-2H3 and PC3 cell lines (MTT asssay, triplicate)	[21]
**47**	spongiain G	diterpene	*Spongia* sp.	Zhanjiang, Guangdong, China	no cytotoxicity against K562, A549, Hela, HT29, SK-BR-3, Huh7, RBL-2H3 and PC3 cell lines (MTT asssay, triplicate)	[21]
**48**	sponalactone	diterpene	*S. officinalis*	Ximao Island, Hainan, China	anti-inflammatory activity toward inhibiting LPS-induced NO production (IC_{50} = 32 ± 4 μM)	[22]
**49**	17-*O*-acetylepispongiatriol	diterpene	*S. officinalis*	Ximao Island, Hainan, China	anti-inflammatory activity toward inhibiting LPS-induced NO production (IC_{50} = 15 ± 3 μM)	[22]
**50**	17-*O*-acetylspongiatriol	diterpene	*S. officinalis*	Ximao Island, Hainan, China	anti-inflammatory activity toward inhibiting LPS-induced NO production (IC_{50} =	[22]

No	Name	Class	Species	Locality	Bioassays	Ref
					12 ± 2 μM)	
**51**	15α,16α-dimethoxy-15,16-dihydroepispongiatriol	diterpene	*S. officinalis*	Ximao Island, Hainan, China	anti-inflammatory activity toward inhibiting LPS-induced NO production (IC_{50} = 22 ± 3 μM)	[22]
**52**	15α-ethoxyepispongiatriol-16(15*H*)-one	diterpene	*S. officinalis*	Ximao Island, Hainan, China	anti-inflammatory activity toward inhibiting LPS-induced NO production (IC_{50} = 12 ± 2 μM)	[22]
**53**	ceylonamide G	diterpene	*Spongia* sp.	Biak, Indonesia	cytotoxicity against DU145 cell line (IC_{50} = 6.9 μM) (MTT asssay, triplicate)	[23]
**54**	ceylonamide H	diterpene	*Spongia* sp.	Biak, Indonesia	cytotoxicity against DU145 cell line (IC_{50} > 100 μM) (MTT asssay, triplicate)	[23]
			Spongia irregularis	Ras Mohamed, Sinai, Egypt	no inhibitory against hepatitis C virus (HCV) (Molecular docking calculations)	[31]
**55**	ceylonamide I	diterpene	*Spongia* sp.	Biak, Indonesia	cytotoxicity against DU145 cell line (IC_{50} > 100 μM) (MTT asssay, triplicate)	[23]
**56**	3β-hydroxyspongia-13(16),14-dien-2-one	diterpene	*Spongia tubulifera*	Mexican Caribbean	cytotoxicity against A549, A2058, HepG2 and MiaPaca-2 cell lines (IC_{50} = 88.1 ± 7.9, 71.4 ± 2.5, 91.3 ± 15.8 and 90.0 ± 44.8 μM, respectively) (MTT asssay, triplicate); no antibacterial activity against *Acinetobacter baumannii*, *Pseudomonas aeruginosa*, *Klebsiella pneumoniae*, and *S. aureus*	[24]
					mitochondrial-mediated neuroprotective properties through direct interaction with cyclophilin D	[25]
**57**	19-dehydroxy-spongian diterpene 17	diterpene	*S. tubulifera*	Mexican Caribbean	no cytotoxicity against A549, A2058, HepG2, MCF7 and MiaPaca-2 cell lines (MTT asssay, triplicate); no antibacterial activity against *A. baumannii*, *P. aeruginosa*, *K. pneumoniae* and *S. aureus*	[24]
					mitochondrial-mediated neuroprotective properties through direct interaction with cyclophilin D	[25]
**58**	9-nor-3-hydroxyspongia-3,13(16),14-trien-2-one	diterpene	*S. tubulifera*	Mexican Caribbean	no cytotoxicity against A549, A2058, HepG2, MCF7 and MiaPaca-2 cell lines (MTT asssay, triplicate); no antibacterial activity against *A. baumannii*, *P. aerugino-*	[24]

No	Name	Class	Species	Locality	Bioassays	Ref
					sa, *K. pneumoniae* and *S. aureus*	
					mitochondrial-mediated neuroprotective properties through direct interaction with cyclophilin D	[25]
**59**	3β,19-dihydroxyspongia-13(16),14-dien-2-one (epispongiadiol)	diterpene	*S. tubulifera*	Mexican Caribbean	cytotoxicity against A549, A2058 and HepG2 cell lines (IC_{50} = 73.7 ± 6.3, 53.9 ± 0.6 and 60.1 ± 5.0 μM, respectively) (MTT asssay, triplicate); no antibacterial activity against *A. baumannii*, *P. aeruginosa*, *K. pneumoniae* and *S. aureus*	[24]
					mitochondrial-mediated neuroprotective properties through direct interaction with cyclophilin D	[25]
**60**	spongian diterpene 17	diterpene	*S. tubulifera*	Mexican Caribbean	no cytotoxicity against A549, A2058, HepG2, MCF7 and MiaPaca-2 cell lines (MTT asssay, triplicate); no antibacterial activity against *A. baumannii*, *P. aeruginosa*, *K. pneumoniae* and *S. aureus*	[24]
					mitochondrial-mediated neuroprotective properties through direct interaction with cyclophilin D	[25]
**61**	ambliol C	diterpene	*S. tubulifera*	Mexican Caribbean	cytotoxicity against A549, A2058, HepG2, MCF7 and MiaPaca-2 cell lines (IC_{50} = 28.3 ± 2.1, 22.9 ± 0.7, 24.3 ± 0.2, 19.9 ± 3.3 and 11.7 ± 0.9 μM, respectively) (MTT asssay, triplicate); no antibacterial activity against *A. baumannii*, *P. aeruginosa*, *K. pneumoniae* and *S. aureus*	[24]
**62**	dinorspongian A	diterpene	*Spongia* sp.	Zhanjiang, Guangdong, China	no cytotoxicity against HeLa, Huh7, RBL-2H3 and PC3 cell lines (MTT assay, triplicate)	[26]
**63**	dinorspongian B	diterpene	*Spongia* sp.	Zhanjiang, Guangdong, China	no cytotoxicity against HeLa, Huh7, RBL-2H3 and PC3 cell lines (MTT assay, triplicate)	[26]
**64**	dinorspongian C	diterpene	*Spongia* sp.	Zhanjiang, Guangdong, China	no cytotoxicity against HeLa, Huh7, RBL-2H3 and PC3 cell lines (MTT assay, triplicate)	[26]

No	Name	Class	Species	Locality	Bioassays	Ref
**65**	dinorspongian D	diterpene	*Spongia* sp.	Zhanjiang, Guangdong, China	no cytotoxicity against HeLa, Huh7, RBL-2H3 and PC3 cell lines (MTT assay, triplicate)	[26]
**66**	dinorspongian E	diterpene	*Spongia* sp.	Zhanjiang, Guangdong, China	no cytotoxicity against HeLa, Huh7, RBL-2H3 and PC3 cell lines (MTT assay, triplicate)	[26]
**67**	dinorspongian F	diterpene	*Spongia* sp.	Zhanjiang, Guangdong, China	no cytotoxicity against HeLa, Huh7, RBL-2H3 and PC3 cell lines (MTT assay, triplicate)	[26]
**68**	epoxynorspongian A	diterpene	*Spongia* sp.	Zhanjiang, Guangdong, China	no cytotoxicity against HeLa, Huh7, RBL-2H3 and PC3 cell lines (MTT assay, triplicate)	[26]
**69**	epoxynorspongian B	diterpene	*Spongia* sp.	Zhanjiang, Guangdong, China	no cytotoxicity against HeLa, Huh7, RBL-2H3 and PC3 cell lines (MTT assay, triplicate)	[26]
**70**	epoxynorspongian C	diterpene	*Spongia* sp.	Zhanjiang, Guangdong, China	no cytotoxicity against HeLa, Huh7, RBL-2H3 and PC3 cell lines (MTT assay, triplicate)	[26]
**71**	epoxynorspongian D	diterpene	*Spongia* sp.	Zhanjiang, Guangdong, China	no cytotoxicity against HeLa, Huh7, RBL-2H3 and PC3 cell lines (MTT assay, triplicate)	[26]
**72**	epoxynorspongian E	diterpene	*Spongia* sp.	Zhanjiang, Guangdong, China	cytotoxicity against RBL-2H3 and PC3 cell lines (IC_{50} = 27.2 and 24.8 μM, respectively), but no cytotoxicity against HeLa and Huh7 cell lines (MTT assay, triplicate)	[26]
**73**	epoxynorspongian F	diterpene	*Spongia* sp.	Zhanjiang, Guangdong, China	no cytotoxicity against HeLa, Huh7, RBL-2H3 and PC3 cell lines (MTT assay, triplicate)	[26]
**74**	17-dehydroxysponalactone	diterpene	*Spongia* sp.	Jeddah, Saudi Arabia	no cytotoxicity against P388, HuCCT, DLD-1 and CCD-966SK cell lines (IC_{50} > 20 μg/mL) (Almar Blue assay, triplicate); anti-inflammatory activity toward inhibiting O_2^- generation and elastase release (IC_{50} = 3.37 ± 0.21 and 4.07 ± 0.60 μM, respectively)	[27]
**75**	18-nor-3,17-dihydroxy-spongia-3,13(16),14-trien-2-one	diterpene	*Spongia* sp.	Jeddah, Saudi Arabia	no cytotoxicity against P388, HuCCT, DLD-1 and CCD-966SK cell lines (IC_{50} > 20	[27]

No	Name	Class	Species	Locality	Bioassays	Ref
					μg/mL) (Almar Blue assay, triplicate); no anti-inflammatory activity toward inhibiting O_2^- generation and elastase release	
			S. officinalis	Shui Niupo Village, Danzhou City, Hainan Province, China	cytotoxicity activities against K562 cell line (IC_{50} = 6.4 μM) (MTT assay, triplicate), but no cytotoxicity against H69AR, ASPC-1 and MDA-MB-231 cell lines (SRB assay, triplicate); signalling inhibition of HIF-1 and Wnt (inhibitory rates 84.7% and 85.2%, respectively), but no signalling inhibition of STAT3/NF-κB, or activation of PPARγ and p53	[28]
**76**	2β,3α,19-Triacetoxy-17-hydroxyspongia-13(16),14-diene	diterpene	*S. officinalis*	Shui Niupo Village, Danzhou City, Hainan Province, China	cytotoxicity activities against K562 cell line (IC_{50} = 7.3 μM) (MTT assay, triplicate), but no cytotoxicity against H69AR, ASPC-1 and MDA-MB-231 cell lines (SRB assay, triplicate); no signalling inhibition of HIF-1, Wnt and STAT3/NF-κB, or activation of PPARγ and p53	[28]
**77**	18-nor-2,17-hydroxyspongia-1,4,13(16),14-quaien-3-one	diterpene	*S. officinalis*	Shui Niupo Village, Danzhou City, Hainan Province, China	no cytotoxicity against K562, H69AR, ASPC-1 and MDA-MB-231 cell lines (MTT assay and SRB assay, triplicate); no signalling inhibition of HIF-1, Wnt and STAT3/NF-κB, or activation of PPARγ and p53	[28]
**78**	3β,17,19-trihydroxyspongia-13(16),14-dien-2-one	diterpene	*S. officinalis*	Shui Niupo Village, Danzhou City, Hainan Province, China	cytotoxicity activities against K562 and H69AR cell lines (IC_{50} = 3.5 and 9.5 μM, respectively) (MTT assay and SRB assay, triplicate), but no cytotoxicity against ASPC-1 and MDA-MB-231 cell lines (SRB assay, triplicate); signalling inhibition of HIF-1 and Wnt (inhibitory rates 88.5% and 87.9%, respectively), but no signalling inhibition of STAT3/NF-κB, or activation of PPARγ and p53	[28]
**79**	2,17,19- trihydroxyspongia-1,13(16),14-trien-3-one	diterpene	*S. officinalis*	Shui Niupo Village, Danzhou City, Hainan Province, China	no cytotoxicity against K562, H69AR, ASPC-1 and MDA-MB-231 cell lines (MTT	[28]

No	Name	Class	Species	Locality	Bioassays	Ref
					assay and SRB assay, triplicate); no signalling inhibition of HIF-1, Wnt and STAT3/NF-κB, or activation of PPARγ and p53	
**80**	3α,17,19-trihydroxyspongia-13(16),14-dien -2-one	diterpene	*S. officinalis*	Shui Niupo Village, Danzhou City, Hainan Province, China	no cytotoxicity against K562, H69AR, ASPC-1 and MDA-MB-231 cell lines (MTT assay and SRB assay, triplicate); no signalling inhibition of HIF-1, Wnt and STAT3/NF-κB, or activation of PPARγ and p53	[28]
**81**	3-nor-spongianone B	diterpene	*S. officinalis*	Shui Niupo Village, Danzhou City, Hainan Province, China	no cytotoxicity against K562, H69AR, ASPC-1 and MDA-MB-231 cell lines (MTT assay and SRB assay, triplicate); no signalling inhibition of HIF-1, Wnt and STAT3/NF-κB, or activation of PPARγ and p53	[28]
**82**	jellynolide A	diterpene	*S. officinalis*	Shui Niupo Village, Danzhou City, Hainan Province, China	no cytotoxicity against K562, H69AR, ASPC-1 and MDA-MB-231 cell lines (SRB assay and MTT assay, triplicate); no anti-polymerase activity against COVID-19 RNA-dependent RNA polymerase; signalling inhibition of TAT3/NF-κB and HIF-1 (inhibitory rates 67.6% and 71.6% at 20 μM, respectively), but no signalling inhibition of Wnt	[29]
**83**	dinorspongiapyridine	diterpene	*Spongia* sp.	Zhanjiang, Guangdong, China	no cytotoxicity against K562, A549, Hela, HT29, SK-BR-3 and Huh7 cell lines (MTT assay, triplicate)	[30]
**84**	19,20-dihydroxyspongia-13(16),14-diene	diterpene	*Spongia irregularis*	Ras Mohamed, Sinai, Egypt	no inhibitory against HCV (Molecular docking calculations)	[31]
**85**	3β-acetoxyspongia-13(16),14-diene	diterpene	*S. irregularis*	Ras Mohamed, Sinai, Egypt	no inhibitory against HCV (Molecular docking calculations)	[31]
**86**	3α-acetoxyspongia-13(16),14-diene	diterpene	*S. irregularis*	Ras Mohamed, Sinai, Egypt	no inhibitory against HCV (Molecular docking calculations)	[31]
**87**	spongianol	diterpene	*Spongia* sp.	Jeddah,Saudi Arabia	no cytotoxicity against Huh7 cell line (MTT asssay, triplicate); no antibacterial activity against *S. aureus*; no an-	[15]

No	Name	Class	Species	Locality	Bioassays	Ref
					ti-inflammatory activity toward inhibiting O_2^- generation and elastase release (IC_{50} > 20 μM)	
**88**	10-hydroxykahukuene B	diterpene	*Spongia* sp.	Jeddah,Saudi Arabia	cytotoxicity against Huh7 cell line (inhibitory rate 17%) (MTT asssay, triplicate); no antibacterial activity against *S. aureus*; no anti-inflammatory activity toward inhibiting O_2^- generation and elastase release (IC_{50} > 20 μM)	[15]
**89**	spongenolactone A	diterpene	*Spongia* sp.	Jeddah, Saudi Arabia	no cytotoxic activity against Huh7 cell line (Resazurin assay, triplicate); antibacterial activity against *S. aureus* (inhibition rate 93% at 200 μM); anti-inflammatory activity toward inhibiting superoxide anion (O_2^-) generation (IC_{50} = 16.5 ± 1.6 μM) and elastase release (IC_{50} > 20 μM)	[32]
**90**	spongenolactone B	diterpene	*Spongia* sp.	Jeddah, Saudi Arabia	no cytotoxic activity against Huh7 cell line (Resazurin assay, triplicate); antibacterial activity against *S. aureus* (inhibition rate 40% at 200 μM); anti-inflammatory activity toward inhibiting superoxide anion (O_2^-) generation (IC_{50} = 13.1 ± 1.3 μM) and elastase release (IC_{50} = 18.6 ± 0.9 μM)	[32]
**91**	spongenolactone C	diterpene	*Spongia* sp.	Jeddah, Saudi Arabia	no cytotoxic activity against Huh7 cell line (Resazurin assay, triplicate); no antibacterial activity against *S. aureus*; anti-inflammatory activity toward inhibiting superoxide anion (O_2^-) generation (IC_{50} = 17.4 ± 1.9 μM) and elastase release (IC_{50} > 20 μM)	[32]
**92**	secodinorspongin A	diterpene	*Spongia* sp.	Jeddah, Saudi Arabia	no cytotoxic activity against Huh7 cell line (Resazurin assay, triplicate); antibacterial activity against *S. aureus* (inhibition rate 75% at 200 μM); no anti-inflammatory activity toward inhibiting superoxide anion (O_2^-) generation (IC_{50} > 20 μM) and elastase release (IC_{50} > 20 μM)	[33]

No	Name	Class	Species	Locality	Bioassays	Ref
**93**	secodinorspongin B	diterpene	*Spongia* sp.	Jeddah, Saudi Arabia	no cytotoxic activity against Huh7 cell line (Resazurin assay, triplicate); no antibacterial activity against *S. aureus*; no anti-inflammatory activity toward inhibiting superoxide anion (O_2^-) generation (IC_{50} > 20 μM) and elastase release (IC_{50} > 20 μM)	[33]
**94**	secodinorspongin C	diterpene	*Spongia* sp.	Jeddah, Saudi Arabia	no cytotoxic activity against Huh7 cell line (Resazurin assay, triplicate); no antibacterial activity against *S. aureus*; no anti-inflammatory activity toward inhibiting superoxide anion (O_2^-) generation (IC_{50} > 20 μM) and elastase release (IC_{50} > 20 μM)	[33]
**95**	secodinorspongin D	diterpene	*Spongia* sp.	Jeddah, Saudi Arabia	no cytotoxic activity against Huh7 cell line (Resazurin assay, triplicate); no antibacterial activity against *S. aureus*; anti-inflammatory activity toward inhibiting superoxide anion (O_2^-) generation (IC_{50} > 20 μM) and elastase release (IC_{50} > 20 μM)	[33]
**96**	sponginolide	diterpene	*Spongia* sp.	Jeddah, Saudi Arabia	no cytotoxic activity against Huh7 cell line (Resazurin assay, triplicate); no antibacterial activity against *S. aureus*; anti-inflammatory activity toward inhibiting superoxide anion (O_2^-) generation (IC_{50} > 20 μM) and elastase release (IC_{50} > 20 μM)	[33]
**97**	/[2]	sesterterpene	*Spongia* sp.	Geoje Island, South Korea	no antagonistic activity against the farnesoid X-activated receptor (FXR) (Co-transfection assay); no cytotoxicity against CV-1 cell line (MTT asssay, triplicate)	[34]
**98**	/	sesterterpene	*Spongia* sp.	Geoje Island, South Korea	no antagonistic activity against FXR (Co-transfection assay); no cytotoxicity against CV-1 cell line (MTT asssay, triplicate)	[34]
**99**	scalarolide acetate	sesterterpene	*Spongia* sp.	Mengalum Island, Sabah, Malaysia	cytotoxic activities against S1T cell line (IC_{50} = 5.16 μg/mL) (WST-8 assay, triplicate)	[35]
**100**	scalarolide	sesterterpene	*Spongia* sp.	Mengalum Island, Sabah, Malay-	cytotoxic activities against S1T cell line	[35]

No	Name	Class	Species	Locality	Bioassays	Ref
				sia	(IC_{50} = 3.93 μg/mL) (WST-8 assay, triplicate)	
**101**	12-*O*-deacetyl-12-*epi*-19-*O*-methylscalarin	sesterterpene	*Spongia* sp.	Mengalum Island, Sabah, Malaysia	c cytotoxic activities against S1T cell line (IC_{50} = 2.31 μg/mL) (WST-8 assay, triplicate)	[35]
**102**	18-hydroxy-19-norscalar-16-en-20-carboxylate	sesterterpene	*Spongia* sp.	Mengalum Island, Sabah, Malaysia	—	[35]
**103**	scalalactam A	sesterterpene	*Spongia* sp.	Tong-Yong City, Korea	no antagonistic activity against FXR (Co-transfection assay); no cytotoxicity against CV-1 cell line (MTT asssay, triplicate)	[36]
**104**	scalalactam B	sesterterpene	*Spongia* sp.	Tong-Yong City, Korea	no antagonistic activity against FXR (Co-transfection assay); no cytotoxicity against CV-1 cell line (MTT asssay, triplicate)	[36]
**105**	scalalactam C	sesterterpene	*Spongia* sp.	Tong-Yong City, Korea	no antagonistic activity against FXR (Co-transfection assay); no cytotoxicity against CV-1 cell line (MTT asssay, triplicate)	[36]
**106**	scalalactam D	sesterterpene	*Spongia* sp.	Tong-Yong City, Korea	no antagonistic activity against FXR (Co-transfection assay); no cytotoxicity against CV-1 cell line (MTT asssay, triplicate)	[36]
**107**	scalarin	sesterterpene	*S. tubulifera*	Mexican Caribbean	no cytotoxicity against A549, A2058, HepG2, MCF7 and MiaPaca-2 cell lines (MTT asssay, triplicate); no antibacterial activity against *A. baumannii*, *P. aeruginosa*, *K. pneumoniae* and *S. aureus*; no antiviral activity against human adenoviruses HAdV5 and HAdV5-GFP	[24]
**108**	scalimide A	sesterterpene	*Spongia* sp.	Philippines	antibacterial activity against *Micrococcus luteus*, *S. aureus* and *Bacillus subtilis* (MIC = 32, 64 and 16 μg/mL, respectively), but no antibacterial activity against *Salmonella typhimurium*, *K. pneumoniae* and *Escherichia coli* (MIC > 128 μg/mL); cytotoxicity against MCF7 cell line (EC_{50} = 25.6 μM)	[37]

No	Name	Class	Species	Locality	Bioassays	Ref
					(MTS asssay, triplicate)	
**109**	scalimide B	sesterterpene	*Spongia* sp.	Philippines	antibacterial activity against and *B. subtilis* (MIC = 128 μg/mL), but no antibacterial activity against *M. luteus*, *S. aureus*, *S. typhimurium*, *K. pneumoniae* and *E. coli* (MIC > 128 μg/mL); cytotoxicity against MCF7 cell line (EC_{50} = 73.9 μM) (MTS assay, triplicate)	[37]
**110**	scalimide C	sesterterpene	*Spongia* sp.	Philippines	antibacterial activity against *M. luteus*, *S. aureus* and *B. subtilis* (MIC = 32, 128 and 4 μg/mL, respectively), but no antibacterial activity against *S. typhimurium*, *K. pneumoniae* and *E. coli* (MIC > 128 μg/mL); cytotoxicity against MCF7 cell line (EC_{50} = 16.0 μM) (MTS asssay, triplicate)	[37]
**111**	scalimide D	sesterterpene	*Spongia* sp.	Philippines	antibacterial activity against *M. luteus*, *S. aureus* and *B. subtilis* (MIC = 32, 64 and 8 μg/mL, respectively), but no antibacterial activity against *S. typhimurium*, *K. pneumoniae* and *E. coli* (MIC > 128 μg/mL); cytotoxicity against MCF7 cell line (EC_{50} = 13.7 μM) (MTS asssay, triplicate)	[37]
**112**	scalimide E	sesterterpene	*Spongia* sp.	Philippines	no antibacterial activity against *M. luteus*, *S. aureus*, *B. subtilis*, *S. typhimurium*, *K. pneumoniae* and *E. coli* (MIC > 128 μg/mL); cytotoxicity against MCF7 cell line (EC_{50} = 26.5 μM) (MTS asssay, triplicate)	[37]
**113**	scalimide F	sesterterpene	*Spongia* sp.	Philippines	antibacterial activity against *Micrococcus luteus*, *S. aureus* and *Bacillus subtilis* (MIC = 64, 128 and 32 μg/mL, respectively), but no antibacterial activity against *Salmonella typhimurium*, *K. pneumoniae* and *Escherichia coli* (MIC > 128 μg/mL); cytotoxicity against MCF7 cell line (EC_{50} = 38.7 μM) (MTS asssay, triplicate)	[37]
**114**	scalimide G	sesterterpene	*Spongia* sp.	Philippines	antibacterial activity against *M. luteus*, *S. aureus* and *B. subtilis* (MIC = 32, 128 and 32	[37]

No	Name	Class	Species	Locality	Bioassays	Ref
					μg/mL, respectively), but no antibacterial activity against *S. typhimurium*, *K. pneumoniae* and *E. coli* (MIC > 128 μg/mL); cytotoxicity against MCF7 cell line (EC_{50} = 31.5 μM) (MTS asssay, triplicate)	
**115**	scalimide H	sesterterpene	*Spongia* sp.	Philippines	antibacterial activity against *M. luteus*, *S. aureus* and *B. subtilis* (MIC = 32, 128 and 8 μg/mL, respectively), but no antibacterial activity against *S. typhimurium*, *K. pneumoniae* and *E. coli* (MIC > 128 μg/mL); cytotoxicity against MCF7 cell line (EC_{50} = 20.7 μM) (MTS asssay, triplicate)	[37]
**116**	scalimide I	sesterterpene	*Spongia* sp.	Philippines	no antibacterial activity against *M. luteus*, *S. aureus*, *B. subtilis*, *S. typhimurium*, *K. pneumoniae* and *E. coli* (MIC > 128 μg/mL); cytotoxicity against MCF7 cell line (EC_{50} = 42.7 μM) (MTS asssay, triplicate)	[37]
**117**	scalimide J	sesterterpene	*Spongia* sp.	Philippines	antibacterial activity against *M. luteus*, *S. aureus*, *B. subtilis*, *S. typhimurium*, *K. pneumoniae* and *E. coli* (MIC = 8, 16, 4, 32, 64 and 64 μg/mL, respectively); cytotoxicity against MCF7 cell line (EC_{50} = 23.3 μM) (MTS asssay, triplicate)	[37]
**118**	scalimide K	sesterterpene	*Spongia* sp.	Philippines	antibacterial activity against *M. luteus*, *S. aureus* and *B. subtilis* (MIC = 16, 32 and 4 μg/mL, respectively), but no antibacterial activity against *S. typhimurium*, *K. pneumoniae* and *E. coli* (MIC > 128 μg/mL); cytotoxicity against MCF7 cell line (EC_{50} = 20.6 μM) (MTS asssay, triplicate)	[37]
**119**	scalimide L	sesterterpene	*Spongia* sp.	Philippines	no antibacterial activity against *M. luteus*, *S. aureus*, *B. subtilis*, *S. typhimurium*, *K. pneumoniae* and *E. coli* (MIC > 128 μg/mL); cytotoxicity against MCF7 cell line (EC_{50} = 42.9 μM) (MTS asssay, triplicate)	[37]
**120**	12-deacetoxy-4-demethyl-11,24-diacetoxy-3,4-methylenedeoxoscalarin	sesterterpene	*Spongia* cf. *agaricina*	Bunaken National Park, North Sulawesi, Indonesia	antibacterial activity against *Arthrobacter crystallopoietes* and *Bacillus megaterium*	[38]

No	Name	Class	Species	Locality	Bioassays	Ref
**121**	isospongiaquinone	meroterpene	*Spongia* sp.	South China Sea	phytoregulating activity for agricultural plants: stimulated the root growth of seedlings of buckwheat and barley	[39]
**122**	ilimaquinone	meroterpene	*Spongia* sp.	South China Sea	phytoregulating activity for agricultural plants: stimulated the root growth of wheat seedlings	[39]
				Son Cha, Lang Co, Thua Thien-Hue city, Vietnam	antibacterial activity against *B. subtilis* and *S. aureus* (MIC = 6.25 and 6.25 μM, respectively), but no antibacterial activity against *K. pneumoniae* and *E. coli* (MIC > 100 μM) (MTT asssay, triplicate)	[42]
					cytotoxicity against A549, MCF7, HeLa and WI-38 cell lines (IC_{50} = 5.9, 8.3, 7.6 and 9.7 μM, respectively)	[43]
**123**	smenoquinone	meroterpene	*Spongia* sp.	South China Sea	no activity on the root growth of the seedlings of plants buckwheat, wheat, soy, and barley	[39]
**124**	18-deoxy-18-formamidodictyoceratin B	meroterpene	*Spongia pertusa*	Yongxing Island, China	no cytotoxicity against U937, A549, HeLa and HepG2 cell lines (CCK-8 assay, triplicate); no CDK-2 affinity (SPR assay)	[40]
					no antifungal activity against *Trichophyton rubrum*, *Trichophyton mentaqrophytes* and *Candida albicans* (Dilution assay)	[47]
**125**	18-deoxy-18-(2-hydroxyacetyl)aminodictyoceratin B	meroterpene	*S. pertusa*	Yongxing Island, China	no cytotoxicity against U937, A549, HeLa and HepG2 cell lines (CCK-8 assay, triplicate); no CDK-2 affinity (SPR assay)	[40]
					no antifungal activity against *T. rubrum*, *T. mentaqrophytes* and *C. albicans* (Dilution assay)	[47]
**126**	dictyoceratin D	meroterpene	*S. pertusa*	Yongxing Island, China	no cytotoxicity against U937, A549, HeLa and HepG2 cell lines (CCK-8 assay, triplicate); no CDK-2 affinity (SPR assay)	[40]
**127**	N-methyl-ent-smenospongine	meroterpene	*S. pertusa*	Yongxing Island, China	no cytotoxicity against U937, A549, HeLa and HepG2 cell lines (CCK-8 assay, triplicate); no CDK-2 affinity (SPR assay)	[40]
**128**	N-methyl-5-epi-smenospongine	meroterpene	*S. pertusa*	Yongxing Island, China	no cytotoxicity against U937, A549, HeLa and HepG2 cell lines (CCK-8 assay, trip-	[40]

No	Name	Class	Species	Locality	Bioassays	Ref
					licate); no CDK-2 affinity (SPR assay)	
**129**	20-demethoxy-20-methylaminodactyloquinone D	meroterpene	*S. pertusa*	Yongxing Island, China	no cytotoxicity against U937, A549, HeLa and HepG2 cell lines (CCK-8 assay, triplicate); CDK-2 affinity (K_d = 4.8 μM) (SPR assay)	[40]
**130**	20-demethoxy-20-methylamino-5-epi-dactyloquinone D	meroterpene	*S. pertusa*	Yongxing Island, China	no cytotoxicity against U937, A549, HeLa and HepG2 cell lines (CCK-8 assay, triplicate); no CDK-2 affinity (SPR assay)	[40]
**131**	20-demethoxy-20-ethoxydactyloquinone E	meroterpene	*S. pertusa*	Yongxing Island, China	n no cytotoxicity against U937, A549, HeLa and HepG2 cell lines (CCK-8 assay, triplicate); no CDK-2 affinity (SPR assay)	[40]
**132**	20-demethoxy-20-ethoxydactyloquinone B	meroterpene	*S. pertusa*	Yongxing Island, China	no cytotoxicity against U937, A549, HeLa and HepG2 cell lines (CCK-8 assay, triplicate); no CDK-2 affinity (SPR assay)	[40]
**133**	20-demethoxy-20-methylaminodactyloquinone B	meroterpene	*S. pertusa*	Yongxing Island, China	no cytotoxicity against U937, A549, HeLa and HepG2 cell lines (CCK-8 assay, triplicate); no CDK-2 affinity (SPR assay)	[40]
**134**	20-demethoxy-20-ethoxycyclospongiaquinone-1	meroterpene	*S. pertusa*	Yongxing Island, China	no cytotoxicity against U937, A549, HeLa and HepG2 cell lines (CCK-8 assay, triplicate); no CDK-2 affinity (SPR assay)	[40]
**135**	yahazunol B	meroterpene	*S. pertusa*	Yongxing Island, China	no cytotoxicity against U937, A549, HeLa and HepG2 cell lines (CCK-8 assay, triplicate); no CDK-2 affinity (SPR assay)	[40]
**136**	5-epi-Smenospongine	meroterpene	*S. pertusa*	Yongxing Island, China	cytotoxicity against U937 cell line (IC_{50} = 2.8, 1.5, and 0.6 μM), but no cytotoxicity against A549, HeLa and HepG2 cell lines (CCK-8 assay, triplicate); no CDK-2 affinity (SPR assay)	[40]
**137**	smenospongine	meroterpene	*S. pertusa*	Yongxing Island, China	cytotoxicity against U937, HeLa and HepG2 cell lines (IC_{50} = 1.5, 8.6 and 6.7 μM, respectively), but no cytotoxicity against A549 cell line (CCK-8 assay, triplicate); no CDK-2 affinity (SPR assay)	[40]
					preferentially eliminates breast cancer stem-like cells via p38/AMPKα pathways	[41]
			Spongia sp.	Son Cha, Lang Co, Thua Thien-Hue city, Vietnam	antibacterial activity against *B. subtilis* and *S. aureus* (MIC = 12.5 and 6.25 μM, respec-	[42]

No	Name	Class	Species	Locality	Bioassays	Ref
					tively), but no antibacterial activity against *K. pneumoniae* and *E. coli* (MIC > 100 μM) (MTT asssay, triplicate)	
					cytotoxicity against A549, MCF7, HeLa and WI-38 cell lines (IC_{50} = 7.8, 8.3, 7.5 and 9.2 μM, respectively)	[43]
**138**	smenospongidine	meroterpene	*S. pertusa*	Yongxing Island, China	cytotoxicity against U937, HeLa and HepG2 cell lines (IC_{50} = 0.60, 5.4 and 3.5 μM, respectively), but no cytotoxicity against A549 cell line (CCK-8 assay, triplicate); no CDK-2 affinity (SPR assay)	[40]
					no antifungal activity against *T. rubrum*, *T. mentaqrophytes* and *C. albicans* (Dilution assay)	[47]
			Spongia sp.	Son Cha, Lang Co, Thua Thien-Hue city, Vietnam	antibacterial activity against *B. subtilis* and *S. aureus* (MIC = 12.5 and 12.5 μM, respectively), but no antibacterial activity against *K. pneumoniae* and *E. coli* (MIC > 100 μM) (MTT asssay, triplicate)	[42]
					cytotoxicity against A549, MCF7, HeLa and WI-38 cell lines (IC_{50} = 4.0, 6.5, 5.0 and 3.0 μM, respectively)	[43]
**139**	langcoquinone A	meroterpene	*Spongia* sp.	Son Cha, Lang Co, Thua Thien-Hue city, Vietnam	antibacterial activity against *B. subtilis* and *S. aureus* (MIC = 12.5 and 12.5 μM, respectively), but no antibacterial activity against *K. pneumoniae* and *E. coli* (MIC > 100 μM) (MTT asssay, triplicate)	[42]
					cytotoxicity against A549, MCF7, HeLa and WI-38 cell lines (IC_{50} = 9.9, 7.9, 8.1 and 8.4 μM, respectively)	[43]
**140**	langcoquinone B	meroterpene	*Spongia* sp.	Son Cha, Lang Co, Thua Thien-Hue city, Vietnam	antibacterial activity against *B. subtilis* and *S. aureus* (MIC = 12.5 and 12.5 μM, respectively), but no antibacterial activity against *K. pneumoniae* and *E. coli* (MIC > 100 μM) (MTT asssay, triplicate)	[42]
					cytotoxicity against A549, MCF7, HeLa and WI-38 cell lines (IC_{50} = 6.2, 8.7, 8.0 and	[43]

No	Name	Class	Species	Locality	Bioassays	Ref
					8.8 μM, respectively)	
**141**	dictyoceratin A	meroterpene	*Spongia* sp.	Son Cha, Lang Co, Thua Thien-Hue city, Vietnam	antibacterial activity against *B. subtilis* and *S. aureus* (MIC = 6.25 and 6.25 μM, respectively), but no antibacterial activity against *K. pneumoniae* and *E. coli* (MIC > 100 μM) (MTT asssay, triplicate)	[42]
					no cytotoxicity against A549, MCF7, HeLa and WI-38 cell lines (IC_{50} > 50 μM)	[43]
			Spongia sp.	Xisha Islands, China	no antifungal activity against *T. rubrum*, *T. mentaqrophytes* and *C. albicans* (Dilution assay)	[44]
**142**	polyfibrospongol B	meroterpene	*Spongia* sp.	Son Cha, Lang Co, Thua Thien-Hue city, Vietnam	no antibacterial activity against *B. subtilis*, *S. aureus*, *K. pneumoniae* and *E. coli* (MIC > 100 μM) (MTT asssay, triplicate)	[42]
					cytotoxicity against MCF7 cell line (IC_{50} = 42.6 μM), but no cytotoxicity against A549, HeLa and WI-38 cell lines (IC_{50} > 50 μM)	[43]
**143**	19-hydroxypolyfibrospongol B	meroterpene	*Spongia* sp.	Son Cha, Lang Co, Thua Thien-Hue city, Vietnam	no antibacterial activity against *B. subtilis*, *S. aureus*, *K. pneumoniae* and *E. coli* (MIC > 100 μM) (MTT asssay, triplicate)	[42]
					cytotoxicity against WI-38 cell line (IC_{50} = 42.1 μM), but no cytotoxicity against A549, HeLa and MCF7 cell lines (IC_{50} > 50 μM)	[43]
**144**	nakijiquinone L	meroterpene	*Spongia* sp.	Son Cha, Lang Co, Thua Thien-Hue city, Vietnam	antibacterial activity against *B. subtilis* and *S. aureus* (MIC = 6.25 and 6.25 μM, respectively), but no antibacterial activity against *K. pneumoniae* and *E. coli* (MIC > 100 μM) (MTT asssay, triplicate)	[42]
			S. pertusa	Yongxing Island, China	antifungal activity against *T. rubrum*, *T. mentaqrophytes* and *C. albicans* (MIC_{50} = 25, 25 and 25 μg/mL, respectively) (Dilution assay)	[47]
**145**	langconol A	meroterpene	*Spongia* sp.	Son Cha, Lang Co, Thua Thien-Hue city, Vietnam	antibacterial activity against *B. subtilis* (MIC = 12.50 μM), but no antibacterial activity against *S. aureus*, *K. pneumoniae*	[43]

No	Name	Class	Species	Locality	Bioassays	Ref
					and *E. coli* (MIC > 100 μM) (MTT asssay, triplicate); no cytotoxicity against A549, MCF7, HeLa and WI-38 cell lines (IC_{50} > 50 μM)	
**146**	langconol B	meroterpene	*Spongia* sp.	Son Cha, Lang Co, Thua Thien-Hue city, Vietnam	no antibacterial activity against *B. subtilis*, *S. aureus*, *K. pneumoniae* and *E. coli* (MIC > 100 μM) (MTT asssay, triplicate); no cytotoxicity against A549, MCF7, HeLa and WI-38 cell lines (IC_{50} > 50 μM)	[43]
**147**	langconol C	meroterpene	*Spongia* sp.	Son Cha, Lang Co, Thua Thien-Hue city, Vietnam	antibacterial activity against *B. subtilis* (MIC = 25.0 μM), but no antibacterial activity against *S. aureus*, *K. pneumoniae* and *E. coli* (MIC > 100 μM) (MTT asssay, triplicate); cytotoxicity against A549, MCF7, HeLa and WI-38 cell lines (IC_{50} = 7.8, 5.0, 7.2 and 8.7 μM, respectively)	[43]
**148**	langcoquinone C	meroterpene	*Spongia* sp.	Son Cha, Lang Co, Thua Thien-Hue city, Vietnam	antibacterial activity against *B. subtilis* and *S. aureus* (MIC = 6.25 and 12.50 μM, respectively), but no antibacterial activity against *K. pneumoniae* and *E. coli* (MIC > 100 μM) (MTT asssay, triplicate); cytotoxicity against A549, MCF7, HeLa and WI-38 cell lines (IC_{50} = 6.6, 9.6, 7.6 and 8.2 μM, respectively)	[43]
**149**	polyfibrospongol A	meroterpene	*Spongia* sp.	Son Cha, Lang Co, Thua Thien-Hue city, Vietnam	no antibacterial activity against *B. subtilis*, *S. aureus*, *K. pneumoniae* and *E. coli* (MIC > 100 μM) (MTT asssay, triplicate); no cytotoxicity against A549, MCF7, HeLa and WI-38 cell lines (IC_{50} > 50 μM)	[43]
**150**	smenospongorine	meroterpene	*Spongia* sp.	Son Cha, Lang Co, Thua Thien-Hue city, Vietnam	antibacterial activity against *B. subtilis* and *S. aureus* (MIC = 12.50 and 25.00 μM, respectively), but no antibacterial activity against *K. pneumoniae* and *E. coli* (MIC > 100 μM) (MTT asssay, triplicate); cytotoxic activities against A549, MCF7, HeLa and WI-38 cell lines (IC_{50} = 6.7, 4.6, 7.6 and 3.5 μM, respectively)	[43]

No	Name	Class	Species	Locality	Bioassays	Ref
				Xisha Islands, China	antifungal activity against *C. albicans* (MIC_{50} = 25 μg/mL), but no antifungal activity against *T. rubrum* and *T. mentaqrophytes* (Dilution assay)	[44]
			S. pertusa	Yongxing Island, China	antifungal activity against *T. rubrum*, *T. mentaqrophytes* and *C. albicans* (MIC_{50} = 100, 50 and 25 μg/mL, respectively) (Dilution assay)	[47]
**151**	smenodiol	meroterpene	*Spongia* sp.	Xisha Islands, China	no antifungal activity against *T. rubrum*, *T. mentaqrophytes* and *C. albicans* (Dilution assay)	[44]
**152**	5-*epi*-smenospongorine	meroterpene	*Spongia* sp.	Xisha Islands, China	antibacterial activity against *T. mentaqrophytes* and *T. rubrrum* (MIC_{80} = 12.5 and 12.5 μg/mL, respectively), but no antifungal activity against *C. albicans* (Dilution assay)	[44]
			S. pertusa	Yongxing Island, China	antifungal activity against *T. rubrum*, *T. mentaqrophytes* and *C. albicans* (MIC_{50} = 12.5, 12.5 and 50 μg/mL, respectively) (Dilution assay)	[47]
**153**	dictyoceratin C	meroterpene	*Spongia* sp.	Xisha Islands, China	no antifungal activity against *T. rubrum*, *T. mentaqrophytes* and *C. albicans* (Dilution assay)	[44]
**154**	*epi*-smenospongidine	meroterpene	*Spongia* sp.	Xisha Islands, China	antibacterial activity against *T. mentaqrophytes* and *T. rubrrum* (MIC_{80} = 25 and 12.5 μg/mL, respectively), but no antifungal activity against *C. albicans* (Dilution assay)	[44]
**155**	langcoquinone D (24-methylsulfinyllancoquinone B)	meroterpene	*Spongia* sp.	Son Cha, Lang Co, Thua Thien-Hue city, Vietnam	antibacterial activity against *B. subtilis* and *S. aureus* (MIC = 12.5 and 25.0 μM, respectively), but no antibacterial activity against *K. pneumoniae* and *E. coli* (MIC > 100 μM) (MTT asssay, triplicate); cytotoxicity against A549, MCF7, HeLa and WI-38 cell lines (IC_{50} = 8.9, 5.9, 8.6 and 5.6 μM, respectively)	[45]
			S. pertusa	Yongxing Island, China	antifungal activity against *T. rubrum*, *T. mentaqrophytes* and *C. albicans* (MIC_{50} = 25,	[47]

No	Name	Class	Species	Locality	Bioassays	Ref
					12.5 and 25 μg/mL, respectively) (Dilution assay)	
**156**	langcoquinone E	meroterpene	*Spongia* sp.	Son Cha, Lang Co, Thua Thien-Hue city, Vietnam	no antibacterial activity against *B. subtilis*, *S. aureus*, *K. pneumoniae* and *E. coli* (MIC > 100 μM) (MTT asssay, triplicate); no cytotoxic activity against A549, MCF7, HeLa and WI-38 cell lines (IC_{50} > 50 μM)	[45]
**157**	langcoquinone F	meroterpene	*Spongia* sp.	Son Cha, Lang Co, Thua Thien-Hue city, Vietnam	no antibacterial activity against *B. subtilis*, *S. aureus*, *K. pneumoniae* and *E. coli* (MIC > 100 μM) (MTT asssay, triplicate); no cytotoxic activity against A549, MCF7, HeLa and WI-38 cell lines (IC_{50} > 50 μM)	[45]
**158**	nakijiquinone E	meroterpene	*S. irregularis*	Ras Mohamed, Sinai, Egypt	no inhibitory against HCV (Molecular docking calculations)	[31]
**159**	nakijiquinone F	meroterpene	*S. irregularis*	Ras Mohamed, Sinai, Egypt	inhibitory against HCV in binding affinity to the active site of NS5B HCV RNA-dependent RNA polymerase (Molecular docking calculations)	[31]
**160**	5-*epi*-isospongiaquinone	meroterpene	*S. irregularis*	Ras Mohamed, Sinai, Egypt	no inhibitory against HCV (Molecular docking calculations)	[31]
**161**	1,4,44-trihydroxy-2-octaprenylbenzene	meroterpene	*S. irregularis*	Ras Mohamed, Sinai, Egypt	no inhibitory against HCV (Molecular docking calculations)	[31]
**162**	2-hexaprenyl-1-4-hydroquinone	meroterpene	*Spongia* sp.	Aegean Sea	cytotoxicity against AGS cell line (IC_{50} = 5.33 μM) but no cytotoxicity against T24 cell line (MTT asssay, triplicate)	[46]
**163**	2-heptaprenyl-1-4-hydroquinone	meroterpene	*Spongia* sp.	Aegean Sea	cytotoxicity against AGS (IC_{50} = 0.994 μM) and T24 (inhibition rate 66.63% at 100 μM) cell lines (MTT asssay, triplicate)	[46]
**164**	2-octaprenyl-1-4-hydroquinone	meroterpene	*Spongia* sp.	Aegean Sea	cytotoxicity against AGS and T24 (inhibition rate 33.41% and 34.38% at 100 μM, respectively) cell lines (MTT asssay, triplicate)	[46]
**165**	2-[24-hydroxy]octaprenyl-1-4-hydroquinone	meroterpene	*Spongia* sp.	Aegean Sea	cytotoxicity against AGS cell line (IC_{50} = 8.09 μM) but no cytotoxicity against T24 cell line (MTT asssay, triplicate)	[46]
**166**	2-hexaprenylmethyl-2-methylchromen-6-	meroterpene	*Spongia* sp.	Aegean Sea	no cytotoxicity against AGS and T24 cell	[46]

No	Name	Class	Species	Locality	Bioassays	Ref
	ol				lines (MTT asssay, triplicate)	
**167**	pelorol A	meroterpene	*S. pertusa*	Yongxing Island, China	no antifungal activity against *T. rubrum*, *T. mentaqrophytes* and *C. albicans* (Dilution assay)	[47]
**168**	*epi*-langconol A	meroterpene	*S. pertusa*	Yongxing Island, China	no antifungal activity against *T. rubrum*, *T. mentaqrophytes* and *C. albicans* (Dilution assay)	[47]
**169**	smenospongic acid	meroterpene	*S. pertusa*	Yongxing Island, China	no antifungal activity against *T. rubrum*, *T. mentaqrophytes* and *C. albicans* (Dilution assay)	[47]
**170**	smenospongiarine	meroterpene	*S. pertusa*	Yongxing Island, China	antifungal activity against *T. rubrum*, *T. mentaqrophytes* and *C. albicans* (MIC_{50} = 25, 12.5 and 50 μg/mL, respectively) (Dilution assay)	[47]
**171**	metachromin X	meroterpene	*Spongia* sp.	Miyako-Sone, Japan	cytotoxicity against HeLa cell line (IC_{50} = 64 μM) (MTT asssay, triplicate); arrest effects on the cell cycle progression of HeLa/Fucci2 cells at S/G2/M phase	[48]
**172**	metachromin Y	meroterpene	*Spongia* sp.	Miyako-Sone, Japan	cytotoxicity against HeLa cell line (IC_{50} = 76 μM) (MTT asssay, triplicate); no arrest effects on the cell cycle progression of HeLa/Fucci2 cells at S/G2/M phase	[48]
**173**	metachromin C	meroterpene	*Spongia* sp.	Miyako-Sone, Japan	cytotoxicity against HeLa cell line (IC_{50} = 53 μM) (MTT asssay, triplicate); arrest effects on the cell cycle progression of HeLa/Fucci2 cells at S/G2/M phase	[48]
**174**	metachromin J	meroterpene	*Spongia* sp.	Miyako-Sone, Japan	cytotoxicity against HeLa cell line (IC_{50} = 73 μM) (MTT asssay, triplicate); no arrest effects on the cell cycle progression of HeLa/Fucci2 cells at S/G2/M phase	[48]
**175**	metachromin T	meroterpene	*Spongia* sp.	Miyako-Sone, Japan	cytotoxicity against HeLa cell line (IC_{50} = 89 μM) (MTT asssay, triplicate); no arrest effects on the cell cycle progression of HeLa/Fucci2 cells at S/G2/M phase	[48]
**176**	demethylfurospongin-4	linear furanoterpenoid	*S. officinalis*	Cortiou and Riou, France	—	[49]
**177**	furofficin	linear furanoterpenoid	*S. officinalis*	Cortiou and Riou, France	—	[49]
**178**	furospongin-1	linear furanoterpenoid	*S. officinalis*	Cortiou and Riou, France	—	[49]

No	Name	Class	Species	Locality	Bioassays	Ref
			Spongia sp.	North Sulawesi, Indonesia	inhibitory against PTP1B, TCPTP and VHR (IC_{50} = 9.9, 9.6 and 11 μM, respectively), but no inhibitory against CD45 (IC_{50} >30 μM); no cytotoxicity against Huh-7 and EJ-1 cell lines (WST-1 asssay, triplicate)	[50]
**179**	spongialactam A	linear furanoterpenoid	*S. officinalis*	Cortiou and Riou, France	—	[49]
**180**	spongialactam B	linear furanoterpenoid	*S. officinalis*	Cortiou and Riou, France	—	[49]
**181**	(-)-untenospongin B	linear furanoterpenoid	*S. officinalis*	Weizhou Island, China	—	[51]
**182**	kurospongin	linear furanoterpenoid	*S. officinalis*	Weizhou Island, China	—	[51]
**183**	fasciculatin acid	linear furanoterpenoid	*S. officinalis*	Weizhou Island, China	—	[51]
**184**	fasciculation	linear furanoterpenoid	*S. officinalis*	Weizhou Island, China	—	[51]
**185**	sponalisolide A	linear furanoterpenoid	*S. officinalis*	Weizhou Island, China	quorum sensing inhibitory activity against *Pseudomonas aeruginosa*	[52]
**186**	sponalisolide B	linear furanoterpenoid	*S. officinalis*	Weizhou Island, China	quorum sensing inhibitory activity against *P. aeruginosa*	[52]
**187**	spongiafuranic acid A	linear furanoterpenoid	*Spongia* sp.	Jeddah, Saudi Arabia	no cytotoxicity against P388, HuCCT, DLD-1 and CCD-966SK cell lines (IC_{50} > 20 μg/mL) (Almar Blue assay, triplicate); no anti-inflammatory activity toward inhibiting O_2^- generation and elastase release (IC_{50} > 10 μM)	[27]
			S. officinalis	Shui Niupo Village, Danzhou City, Hainan Province, China	no cytotoxicity against K562, H69AR, ASPC-1 and MDA-MB-231 cell lines (SRB assay and MTT assay, triplicate); no signalling inhibition of TAT3/NF-*κ*B, HIF-1 and Wnt	[29]
**188**	spongiafuranohydroxamic acid A	linear furanoterpenoid	*Spongia* sp.	Jeddah, Saudi Arabia	no cytotoxicity against P388, HuCCT, DLD-1 and CCD-966SK cell lines (IC_{50} > 20 μg/mL) (Almar Blue assay, triplicate); no anti-inflammatory activity toward inhibiting O_2^- generation and elastase release (IC_{50} > 10 μM)	[27]
**189**	16-*epi*-irciformonin G	linear furanoterpenoid	*Spongia* sp.	Jeddah, Saudi Arabia	no cytotoxicity against P388, HuCCT, DLD-1 and CCD-966SK cell lines (IC_{50} > 20 μg/mL) (Almar Blue assay, triplicate); no anti-inflammatory activity toward inhib-	[27]

No	Name	Class	Species	Locality	Bioassays	Ref
					iting O_2^- generation and elastase release (IC_{50} > 10 μM)	
**190**	(±)-sponalisolide B	linear furanoterpenoid	*Spongia* sp.	Jeddah, Saudi Arabia	no cytotoxicity against P388, HuCCT, DLD-1 and CCD-966SK cell lines (IC_{50} > 20 μg/mL) (Almar Blue assay, triplicate); anti-inflammatory activity toward inhibiting O_2^- generation (IC_{50} = 5.31 ± 1.52 μM) but no inhibition against elastase release	[27]
			S. officinalis	Shui Niupo Village, Danzhou City, Hainan Province, China	no cytotoxicity against K562, H69AR, ASPC-1 and MDA-MB-231 cell lines (SRB assay and MTT assay, triplicate); no signalling inhibition of TAT3/NF-κB, HIF-1 and Wnt	[29]
**191**	(±)-pokepola ester B	linear furanoterpenoid	*S. officinalis*	Shui Niupo Village, Danzhou City, Hainan Province, China	cytotoxicity against MDA-MB-231, K562 and ASPC-1 cell lines (IC_{50} = 17.4, 28.0 and 11.0 μM, respectively), but no cytotoxicity against H69AR cell line (SRB assay and MTT assay, triplicate); no signalling inhibition of TAT3/NF-κB, HIF-1 and Wnt	[29]
**192**	(±)-pokepola ester C	linear furanoterpenoid	*S. officinalis*	Shui Niupo Village, Danzhou City, Hainan Province, China	no cytotoxicity against K562, H69AR, ASPC-1 and MDA-MB-231 cell lines (SRB assay and MTT assay, triplicate); signalling inhibition of TAT3/NF-κB, HIF-1 and Wnt	[29]
**193**	(±)-pokepola ester D	linear furanoterpenoid	*S. officinalis*	Shui Niupo Village, Danzhou City, Hainan Province, China	cytotoxicity against MDA-MB-231 cell lines (IC_{50} = 23.6 μM), but no cytotoxicity against K562, H69AR and ASPC-1 cell lines (SRB assay and MTT assay, triplicate); no signalling inhibition of TAT3/NF-κB, HIF-1 and Wnt	[29]
**194**	(±)-pokepola ester E	linear furanoterpenoid	*S. officinalis*	Shui Niupo Village, Danzhou City, Hainan Province, China	no cytotoxicity against K562, H69AR, ASPC-1 and MDA-MB-231 cell lines (SRB assay and MTT assay, triplicate); no signalling inhibition of TAT3/NF-κB, HIF-1 and Wnt	[29]
**195**	(±)-sponalisolide C	linear furanoterpenoid	*S. officinalis*	Shui Niupo Village, Danzhou City, Hainan Province, China	no cytotoxicity against K562, H69AR, ASPC-1 and MDA-MB-231 cell lines (SRB	[29]

No	Name	Class	Species	Locality	Bioassays	Ref
					assay and MTT assay, triplicate); no signalling inhibition of TAT3/NF-κB, HIF-1 and Wnt	
**196**	(-)-sponalisolide D	linear furanoterpenoid	*S. officinalis*	Shui Niupo Village, Danzhou City, Hainan Province, China	no cytotoxicity against K562, H69AR, ASPC-1 and MDA-MB-231 cell lines (SRB assay and MTT assay, triplicate); no signalling inhibition of TAT3/NF-κB, HIF-1 and Wnt	[29]
**197**	stigmsta-4,6,8(14),22-tetraen-3-one	steroid	*Spongia* sp.	Xisha Islands, China	no antifungal activity against *C. albicans*, *T. rubrum* and *T. mentaqrophytes* (Dilution assay)	[44]
**198**	3-oxo-4,6,8(14)-triunsaturated steroid	steroid	*Spongia* sp.	Xisha Islands, China	no antifungal activity against *C. albicans*, *T. rubrum* and *T. mentaqrophytes* (Dilution assay)	[44]
**199**	ergosta-4,6,8(14),22-tetraen-3-one	steroid	*Spongia* sp.	Xisha Islands, China	antifungal activity against *T. rubrum* (MIC_{80} = 25 μg/mL), but no antifungal activity against *C. albicans* and *T. mentaqrophytes* (Dilution assay)	[44]
**200**	cholesta-7-ene-3β,5α-diol-6-one	steroid	*Spongia* sp.	Jeddah, Saudi Arabia	no cytotoxicity against P388, HuCCT, DLD-1 and CCD-966SK cell lines (IC_{50} > 20 μg/mL) (Almar Blue assay, triplicate); no anti-inflammatory activity toward inhibiting O_2^- generation and elastase release	[27]
**201**	3β,5α,9α-trihydroxy-24*S*-ethylcholest-7-en-6-one	steroid	*Spongia* sp.	Jeddah, Saudi Arabia	no cytotoxicity against the Huh7 cell line (MTT asssay, triplicate); no antibacterial activity against *S. aureus*; no anti-inflammatory activity toward inhibiting O_2^- generation and elastase release (IC_{50} > 20 μM)	[15]
**202**	(22*E*,24*S*)-ergosta-7,22-dien-3β,5α-diol-6,5-olide	steroid	*Spongia* sp.	Jeddah, Saudi Arabia	no cytotoxicity against the Huh7 cell line (MTT asssay, triplicate); no antibacterial activity against *S. aureus*; no anti-inflammatory activity toward inhibiting O_2^- generation and elastase release (IC_{50} > 20 μM)	[15]
**203**	/	alkaloid	*Spongia* sp.	Tong-Yong City, Korea	no antagonistic activity against FXR (Co-transfection assay); no cytotoxicity	[53]

No	Name	Class	Species	Locality	Bioassays	Ref
					against CV-1 cell line (MTT asssay, triplicate)	
**204**	/	alkaloid	*Spongia* sp.	Tong-Yong City, Korea	no antagonistic activity against FXR (Co-transfection assay); no cytotoxicity against CV-1 cell line (MTT asssay, triplicate)	[53]
**205**	1,3,7-trimethylguanine	alkaloid	*S. irregularis*	Ras Mohamed, Sinai, Egypt	no inhibitory against HCV (Molecular docking calculations)	[31]
**206**	thymidine	alkaloid	*S. irregularis*	Ras Mohamed, Sinai, Egypt	no inhibitory against HCV (Molecular docking calculations)	[31]
			Spongia sp.	Jeddah, Saudi Arabia	antibacterial activity against *S. aureus* (inhibition rate 89% at 200 μM); no cytotoxicity against Huh7 cell line (MTT asssay, triplicate); no anti-inflammatory activity toward inhibiting O_2^- generation and elastase release (IC_{50} > 20 μM)	[15]
**207**	1*H*-indazole	alkaloid	*S. irregularis*	Ras Mohamed, Sinai, Egypt	no inhibitory against HCV (Molecular docking calculations)	[31]
			Spongia sp.	Zhanjiang, Guangdong, China	—	[30]
**208**	spongiacysteine	alkaloid	*S. irregularis*	Ras Mohamed, Sinai, Egypt	no inhibitory against HCV (Molecular docking calculations)	[31]
**209**	dysidamide	alkaloid	*Spongia* sp.	Jeddah, Saudi Arabia	no cytotoxicity against Huh7 cell line (MTT asssay, triplicate); no antibacterial activity against *S. aureus*; anti-inflammatory activity toward inhibiting elastase release (IC_{50} = 17.23 ± 2.45 μM), but no anti-inflammatory activity toward inhibiting O_2^- generation (IC_{50} > 20 μM)	[15]
**210**	7,7,7-trichloro-3-hydroxy-2,2,6-trimethyl-4-(4,4,4-trichloro-3-methyl-1-oxobu-tylamino)-heptanoic acid methyl ester	alkaloid	*Spongia* sp.	Jeddah, Saudi Arabia	no cytotoxicity against the Huh7 cell line (MTT asssay, triplicate); no antibacterial activity against *S. aureus*; anti-inflammatory activity toward inhibiting elastase release (IC_{50} = 14.60 ± 2.24 μM), but no anti-inflammatory activity toward inhibiting O_2^- generation (IC_{50} > 20 μM)	[15]
**211**	1*H*-indole-3-carbaldehyde	alkaloid	*Spongia* sp.	Zhanjiang, Guangdong, China	—	[30]
**212**	1*H*-indole-3-carboxylic acid	alkaloid	*Spongia* sp.	Zhanjiang, Guangdong, China	—	[30]

No	Name	Class	Species	Locality	Bioassays	Ref
**213**	1*H*-indole-3-carboxylic acid acetate	alkaloid	*Spongia* sp.	Zhanjiang, Guangdong, China	—	[30]
**214**	spongimide A	alkaloids	*Spongia* sp.	Zhanjiang, Guangdong, China	no cytotoxicity against K562 cell line (CCK-8 asssay, triplicate); no antibacterial antibacterial activity against *Salmonella paratyphi* and *E. coli*	[54]
**215**	spongimide B	alkaloid	*Spongia* sp.	Zhanjiang, Guangdong, China	no cytotoxicity against K562 cell line (CCK-8 asssay, triplicate); no antibacterial antibacterial activity against *Salmonella paratyphi* and *E. coli*	[54]
**216**	(*S*)-tetrahydro-1*H*-pyrrolo[1,2-c]imidazole -1,3(2*H*)-dione	alkaloid	*Spongia* sp.	Zhanjiang, Guangdong, China	—	[54]
**217**	(*S*)-5-isopropylhydantoin	alkaloid	*Spongia* sp.	Zhanjiang, Guangdong, China	—	[54]
**218**	*N*-(4-hydroxyphenethyl)succinimide	alkaloid	*Spongia* sp.	Zhanjiang, Guangdong, China	—	[54]
**219**	methyl hematinate	alkaloid	*Spongia* sp.	Zhanjiang, Guangdong, China	—	[54]
**220**	L-pyroglutamic acid methyl ester	alkaloid	*Spongia* sp.	Zhanjiang, Guangdong, China	—	[54]
**221**	3,5-dihydroxyfuran-2(5*H*)-one	others	*S. irregularis*	Ras Mohamed, Sinai, Egypt	no inhibitory against HCV (Molecular docking calculations)	[31]
**222**	(*Z*)-3-methyl-9-oxodec-2-enoic acid	others	*Spongia* sp.	Jeddah, Saudi Arabia	no antibacterial activity against *S. aureu*); no cytotoxicity against Huh7 cell line (MTT asssay, triplicate); no an-ti-inflammatory activity toward inhibiting O_2^- generation and elastase release (IC_{50} > 20 μM)	[15]

[1] '—' indicates no bioassay was reported in the reference.

[2] '/' indicates no compound name was given in the reference.
